# Three-dimensional computed tomography mapping techniques in the morphometric analysis of AO/OTA 33A and 33C distal femoral fractures: a retrospective single-center study

**DOI:** 10.3389/fbioe.2023.1162214

**Published:** 2023-06-15

**Authors:** Song Chen, Zhenxin Zheng, Jinku Guo, Shengkun Hong, Weijun Zhou, Jun Xie, Wei Wang

**Affiliations:** Department of Orthopedics, The Quzhou Affiliated Hospital of Wenzhou Medical University, Quzhou People’s Hospital, Quzhou, Zhejiang Province, China

**Keywords:** distal femoral fractures, fracture line, comminuted area, morphometric analysis, three dimension (3D)

## Abstract

**Purpose:** Complex distal femoral fractures involve a challenging set of considerations that must be known to provide optimal management. This study aimed to determine the location and frequency of fracture lines and comminution zones in AO/OTA types 33A and 33C distal femoral fractures using three-dimensional computed tomography mapping.

**Methods:** Seventy-four consecutive eligible patients were included. Fracture fragments for each patient were reconstructed, virtually reduced, and adjusted to match the distal femoral template. Then, all fracture lines and comminuted areas were extracted in transparent mode, and corresponding heat maps were constructed. Finally, these maps, along with the quantitative analysis findings of the counts and volumes of each fragment, were used to summarize the characteristics of the fractures.

**Results:** Thirty-four females and 40 males [average age, 58 years (range, 18–92 years)] presented with a distal femoral fracture. There were 53 AO/OTA type 33A fractures, and 21 AO/OTA type 33C fractures. These two patterns differed significantly on fracture fragment count, comminuted zone fracture fragment count, and mean comminuted zone fracture fragment volume (*p* < 0.05). Most of the fracture line heat zones were in the femoral epiphysis, intercondylar notch of the femur, and patellofemoral joint. The comminuted area heat regions were mostly found on the lateral, anterior, and posterior femoral diaphysis, with less involvement on the medial side.

**Conclusion:** Our findings may serve as a guide for the surgical approach selection of complex distal femur fractures, the placement strategy of the internal fixation, and the optimization of the osteotomy plan for biomechanical studies.

## Introduction

Distal femoral fractures are morphologically complex, accounting for 0.5% of all fractures and 6% of femoral fractures (Martinet et al., 2000; Pietu et al., 2014; Elsoe et al., 2018). The patients have a bimodal distribution, with younger patients often caused by high-energy trauma and older osteoporotic patients with a history of low-energy trauma, especially in postmenopausal women (Martinet et al., 2000; Elsoe et al., 2018). With the increasing aging of the global population, the incidence of distal femoral fractures in the elderly is increasing annually (Martinet et al., 2000; Elsoe et al., 2018), a trend that may lead to the emergence of new fracture patterns and associated complications. Currently, studies related to distal femoral fractures have focused on internal implant fixation techniques and postoperative complication management. Among them, adverse outcomes after distal femoral comminuted metaphyseal bone surgery are frequently reported, such as delayed or non-union of the fracture, joint stiffness, plate fracture, and loss of alignment after anatomical reduction (Henderson et al., 2011; Kim et al., 2017; Bologna et al., 2020; Chandra Vemulapalli et al., 2022; Kerr et al., 2022). However, there are few studies on the morphological systems of distal femoral fractures, and the lack of exploration of the morphological characteristics of this type of fracture may hinder the optimization of surgical fixation strategies and the design of internal implants for these complex fractures. In the face of this dilemma, there is an urgent need to conduct morphological studies related to distal femoral fractures in order to provide a reference for the classification of these fractures and the updating of treatment strategies.

In recent years, the diagnostic efficacy of complex intra-articular fractures has substantially increased due to the application and ongoing development of computed tomography (CT) imaging technologies. Now, it is frequently utilized for the identification of distal femoral fractures and preoperative planning, hence minimizing the fracture leakage rate, and facilitating the accurate execution of surgery (Nork et al., 2005; Gwathmey et al., 2010; Victor and Premanathan, 2013; Zeng et al., 2016). It also gives technical support for the research of the morphology of distal femoral fractures. Using a CT-based fracture mapping technique, Xie et al. (Xie et al., 2020) investigated Hoffa fracture morphology and discovered that these fractures were more prevalent in the lateral femoral condyle and that fracture lines and comminuted areas were predominantly distributed centripetally. These findings provide useful information for the diagnosis and treatment aspects of Hoffa fractures, but there are few studies on other types of distal femoral fractures. In light of these findings, we propose to use the three dimensional (3D) fracture mapping technique (Armitage et al., 2009; Cole et al., 2013) to depict the location and frequency of fracture lines and comminuted areas in AO/OTA 33A and 33C fractures of the distal femur, based on previous studies, in order to enhance the understanding of these two fracture patterns and thus provide more reference data for future biomechanical studies, surgical treatment, and internal fixation device design.

## Materials and methods

### Subjects

We retrospectively retrieved the clinical and imaging data of patients admitted with distal femur fractures from December 2013 to December 2020. The inclusion criteria for cases were: (i) preoperative diagnosis of a distal femoral fracture and intraoperative confirmation; (ii) unilateral, closed fracture; (iii) age greater than or equal to 18 years; (iv) complete imaging data; and (v) AO/OTA classification (Meinberg et al., 2018) of a 33A or 33C distal femoral fracture. Exclusion criteria: (i) open or pathological fracture; (ii) imaging data not meeting reconstruction criteria; (iii) distal femoral deformity or history of previous surgery. According to the above inclusion and exclusion criteria, of the 86 patients with distal femoral fractures, two (2.3%) were excluded because of an age discrepancy, and 10 (11.9%) were excluded because of AO/OTA 33B distal femoral fractures. Finally, a total of 74 patients with distal femoral fractures were included in this study. The institutional ethics committee of our institution approved this research, and individual consent for this retrospective analysis was waived because all data were deidentified.

## Fracture mapping

**FIGURE 1:**
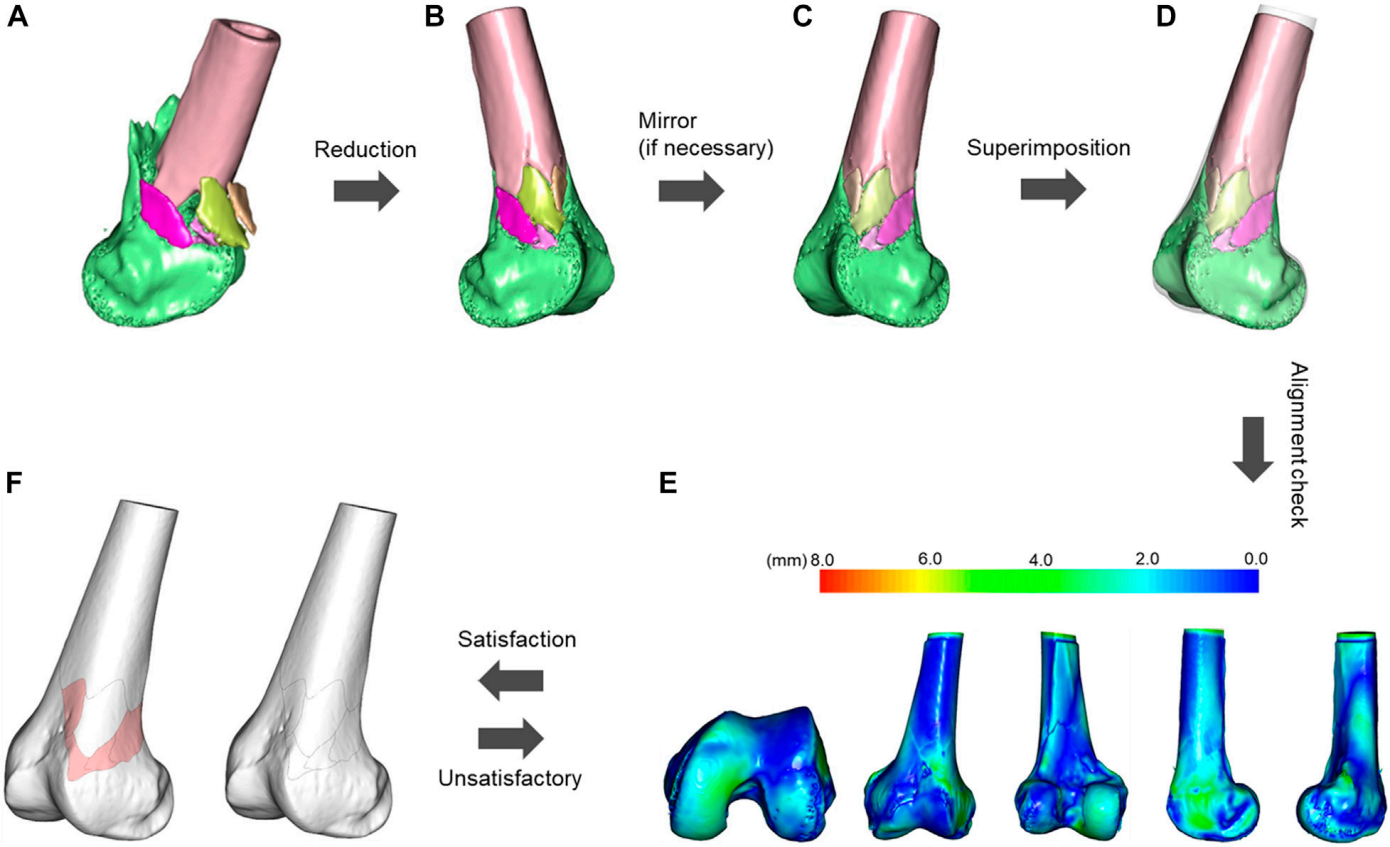
Flowchart of distal femoral fracture line mapping. **(A)** Semi-automatic segmentation, extraction, and reconstruction of the fracture fragments. **(B)** Virtual reduction of the fracture fragments. **(C)** If the fracture model does not match the template laterally, it needs to be mirrored. **(D)** Alignment of the fracture model with the template in transparent mode based on distinct anatomical features. **(E)** Measure the distance between the fracture model and the template to ensure that the maximum distance between the models is within 5 mm, and if this is not the case, fine-tune the fracture model until the conditions are met. **(F)** Adjust the appropriate transparency and copy the outline of each fracture fragment of the fracture model using free curves along the surface of the template in a three-dimensional view, extracting the fracture line and the comminuted area (pink area). The fracture area is defined as a region containing less than 1 cm^3^ of fracture fragments.

**FIGURE 2:**
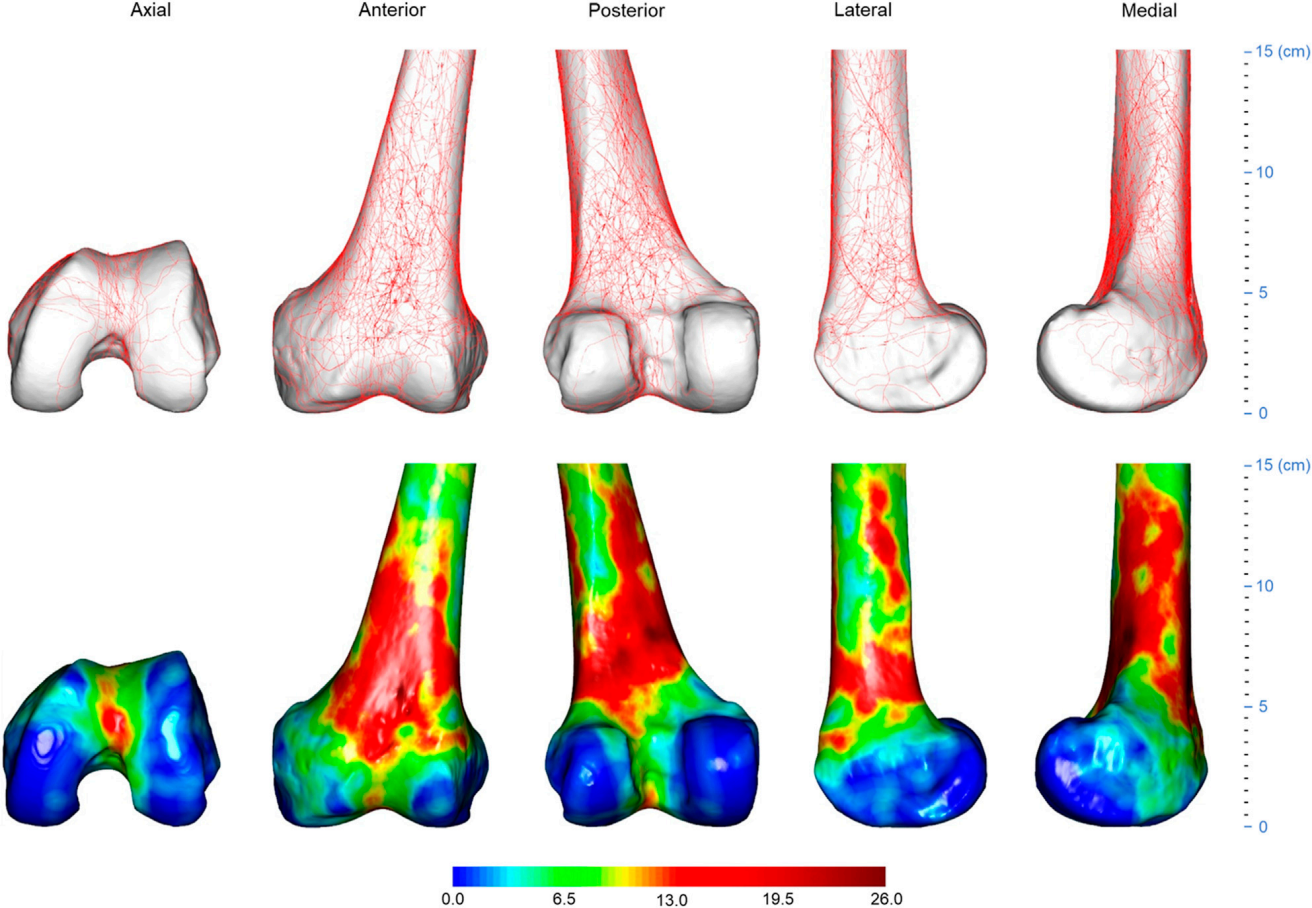
Three-dimensional (3D) spatial distribution of distal femoral fracture lines (*n* = 74). All fracture lines extracted were superimposed on a standard 3D distal femur model, as shown in the top row. The fracture line frequency distribution was then displayed using the heat map technique, as shown in the bottom row. The gradient hues each correlate to a distinct frequency value. The frequency value of a color is larger the closer it is to dark red. The closer a color is approaching dark blue, the closer its frequency value tends to approach zero.

**FIGURE 3:**
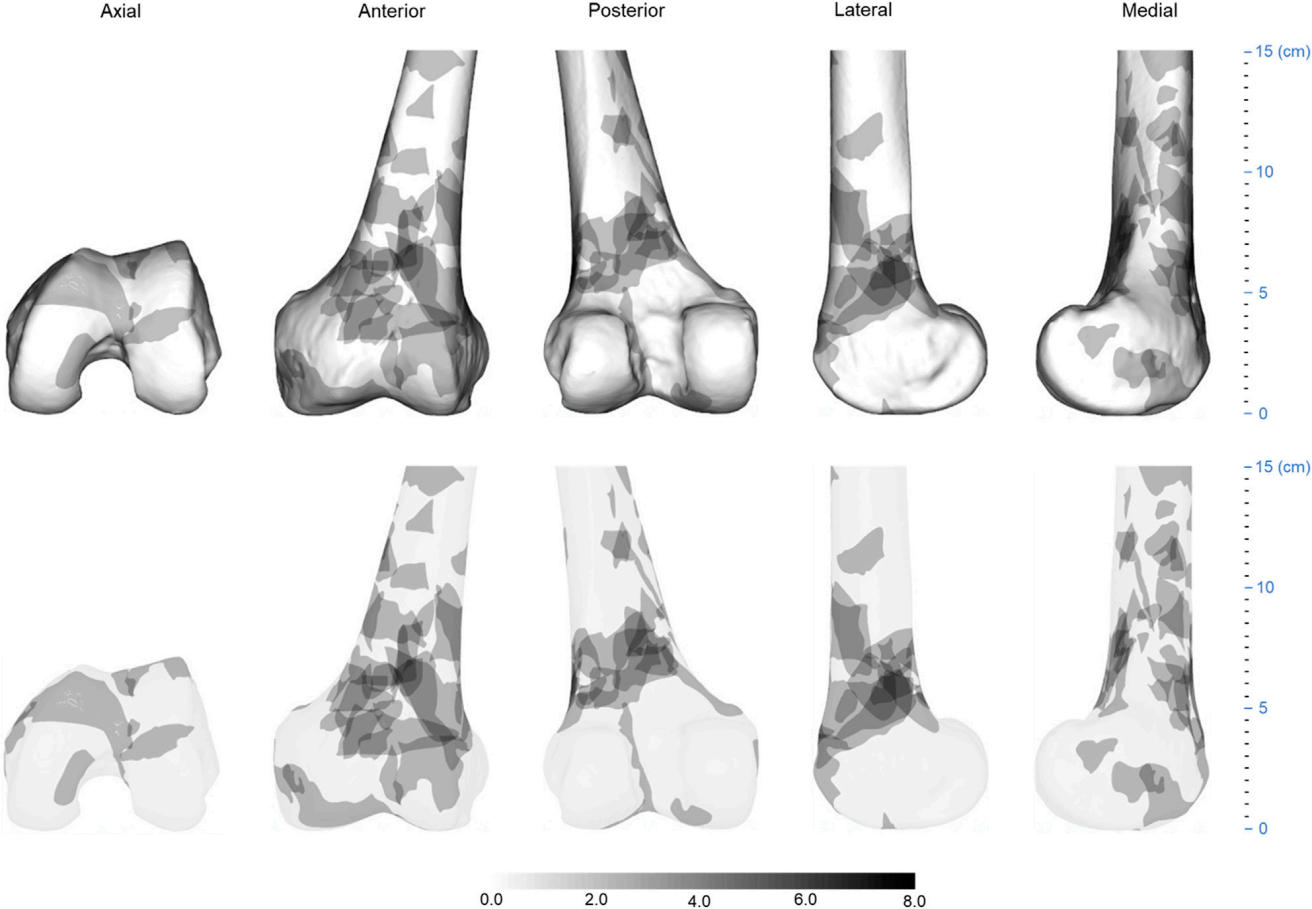
Three-dimensional (3D) spatial distribution of the distal femur fracture comminution area (*n* = 74). All extracted comminuted regions were superimposed on a standard 3D distal femur model, as shown in the top row. The heat map technique was then used to display the distribution of comminuted regions, as shown in the bottom row. The gradient hues each correlate to a distinct frequency value. The frequency value of a color increases as it gets closer to black. The closer a color is to white, the closer its frequency value tends to approach zero.

**FIGURE 4:**
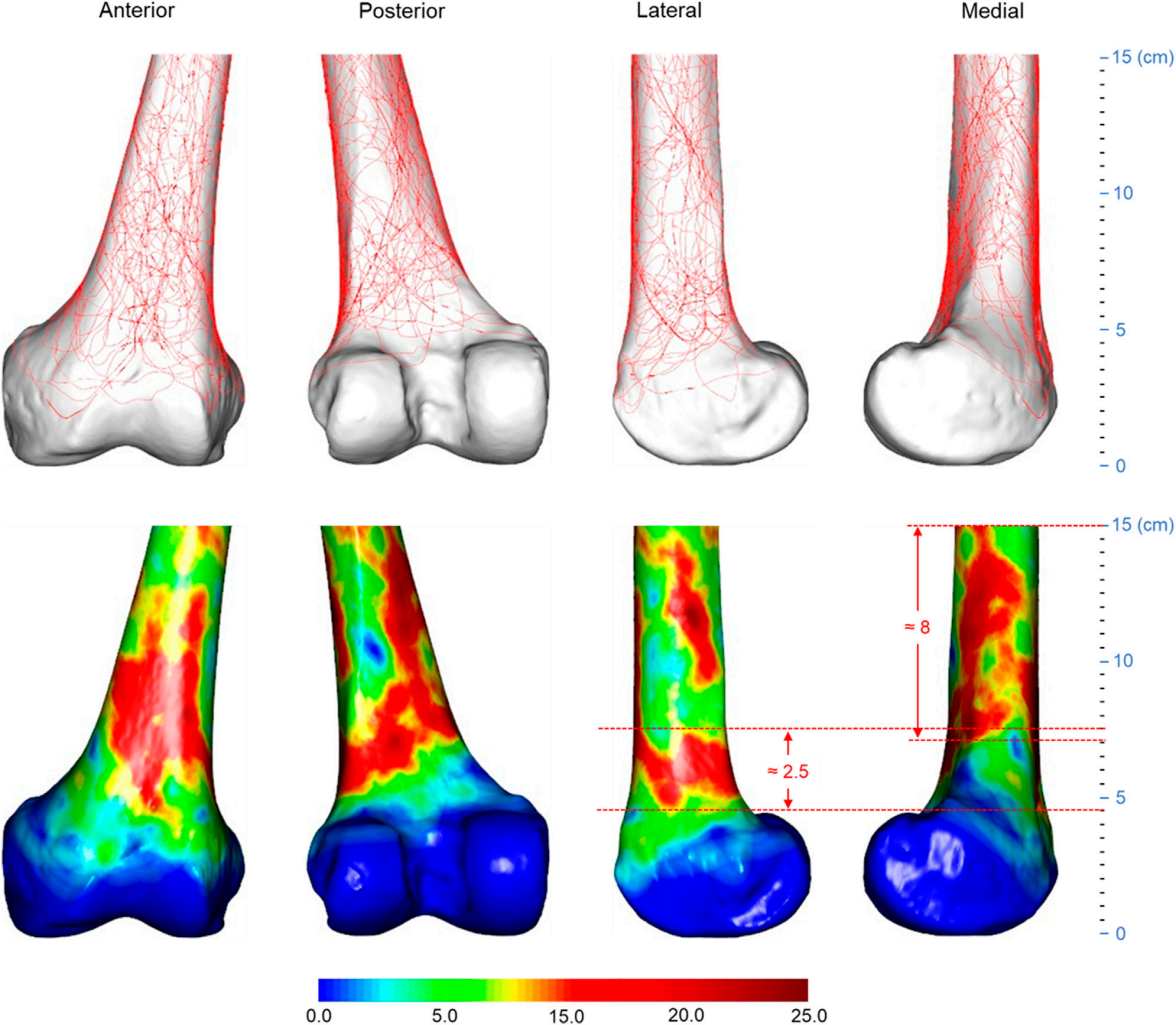
Three-dimensional spatial distribution of AO/OTA 33A distal femoral fracture lines (*n* = 53). The height of the lateral heat zone is roughly 2.5 cm, and the medial heat zone is roughly 8 cm. The distance between the joint line and the bottom edge of the lateral heat zone is roughly 4.5 cm. Abbreviations: AO/OTA, Arbeitsgemeinschaftfür Osteosynthesefragen Foundation and the Orthopaedic Trauma Association.

**FIGURE 5:**
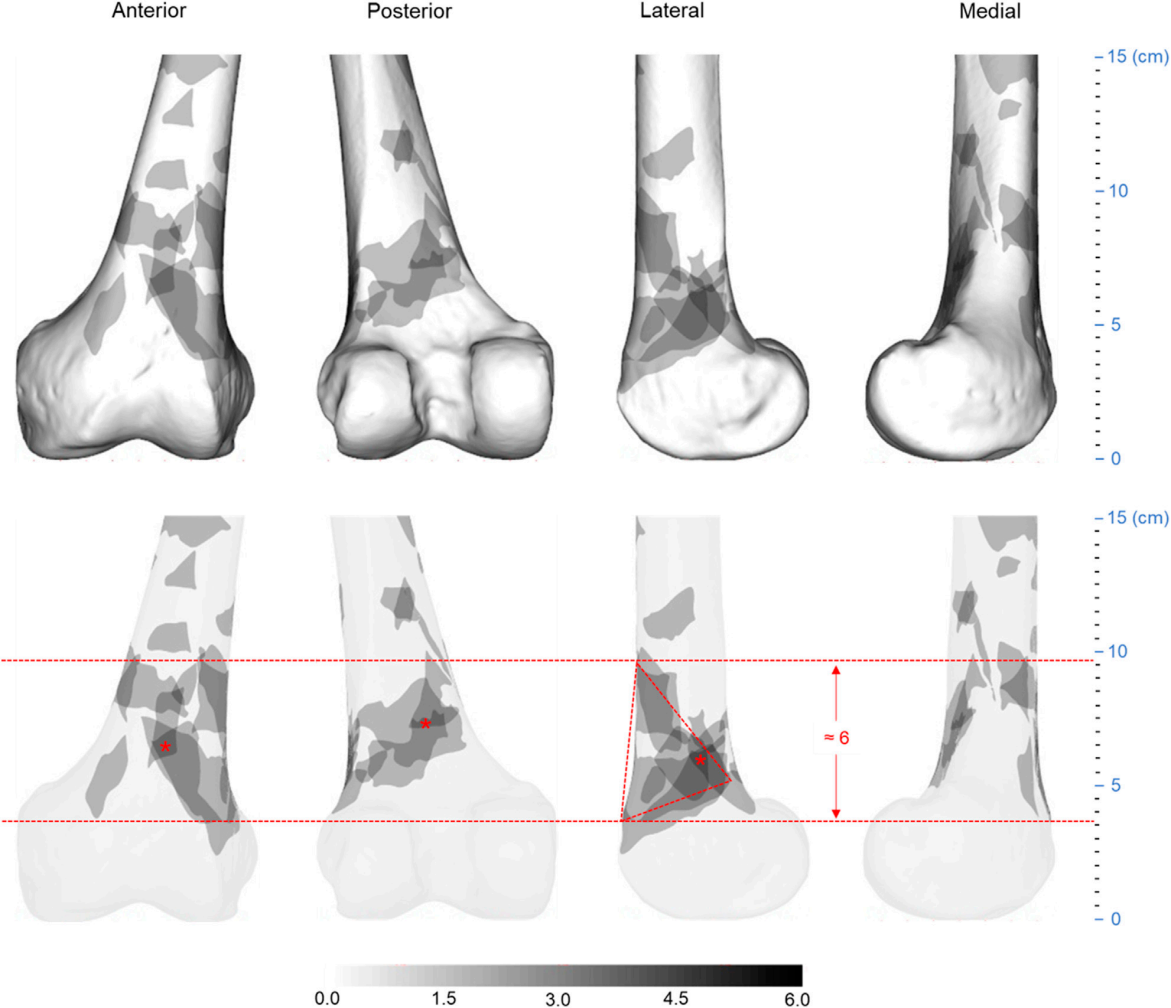
Three-dimensionalspatial distribution of AO/OTA 33A distal femur fracture comminution area (*n* = 53). The medial heat zone of the model is approximately 6 cm in vertical height and is distributed in an approximate triangle from anterior to posterior. The “*” denotes the most concentrated portion of the comminuted area, which is centripetally dispersed. Abbreviations: AO/OTA, Arbeitsgemeinschaftfür Osteosynthesefragen Foundation and the Orthopaedic Trauma Association.

**FIGURE 6:**
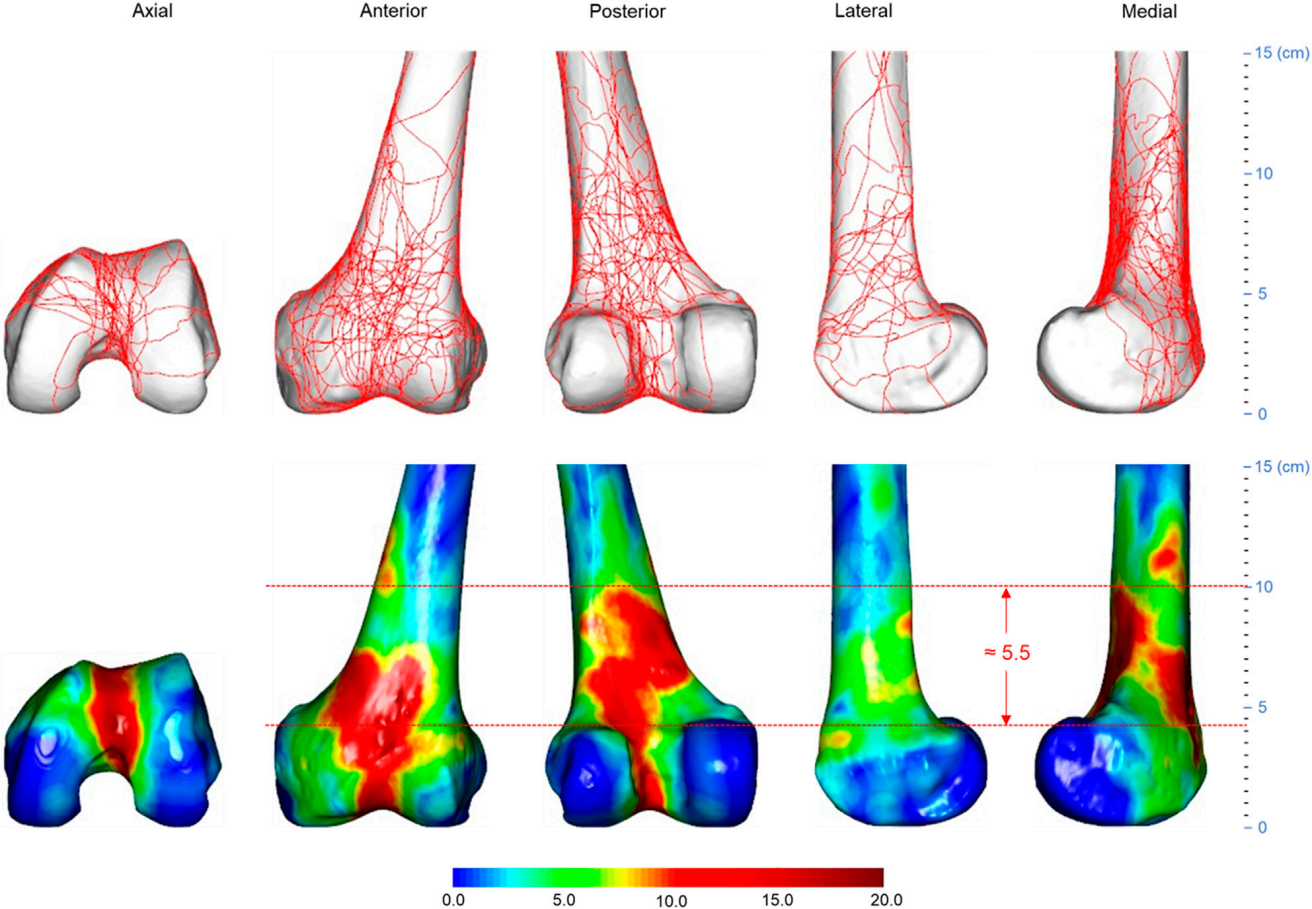
Three-dimensional spatial distribution of AO/OTA 33C distal femoral fracture lines (*n* = 21). The height of the medial heat zone is roughly 5.5 cm. The distance between the joint line and the bottom edge of the medial heat zone is roughly 4 cm. Abbreviations: AO/OTA, Arbeitsgemeinschaftfür Osteosynthesefragen Foundation and the Orthopaedic Trauma Association.

**FIGURE 7:**
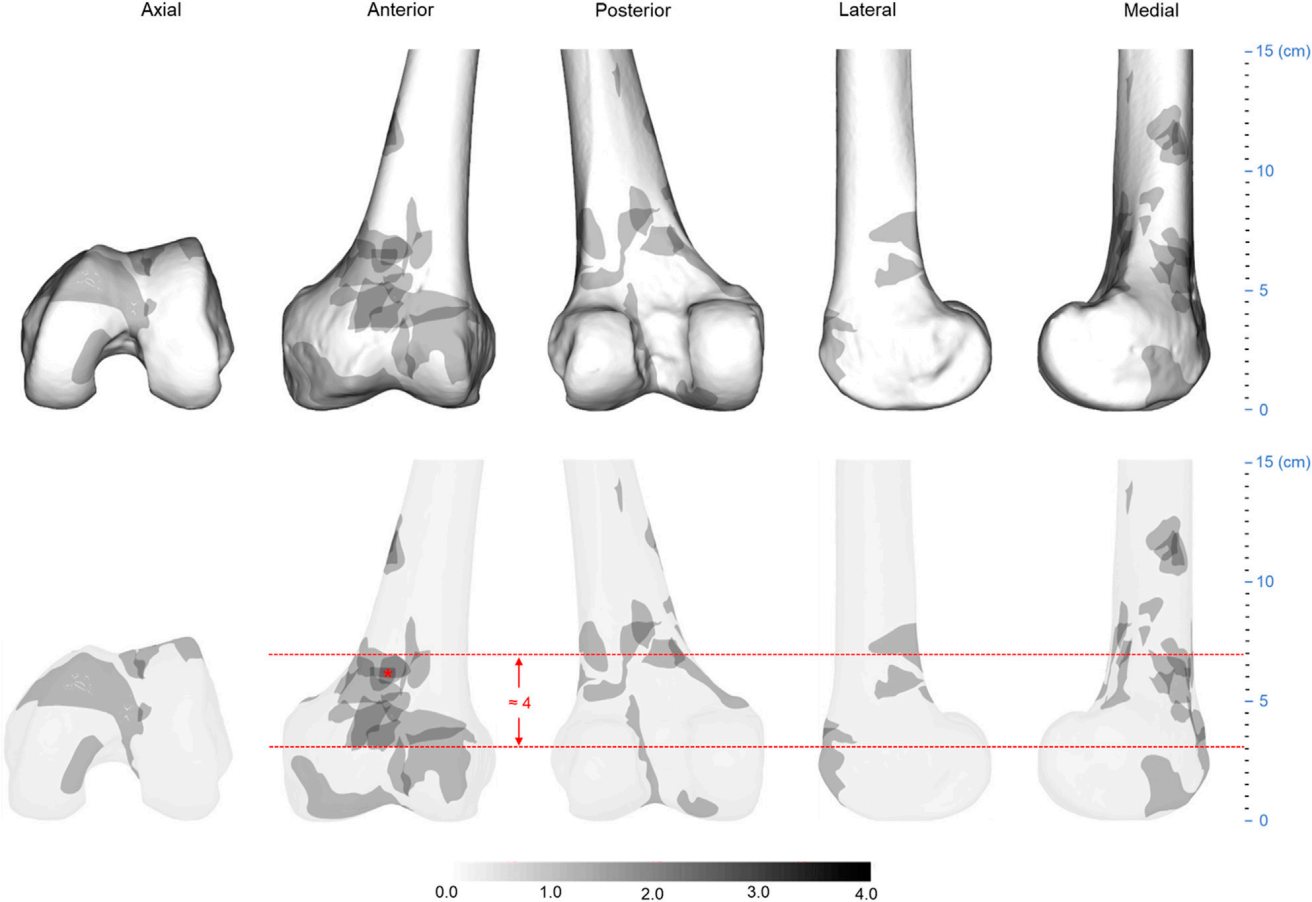
Three-dimensional spatial distribution of AO/OTA 33C distal femur fracture comminution area (*n* = 21). The height of the heat zone on the anterior medial side of the model is approximately 4 cm. The “*” indicates the most concentrated part of the comminuted area. Abbreviations: AO/OTA, Arbeitsgemeinschaftfür Osteosynthesefragen Foundation and the Orthopaedic Trauma Association.

This study utilized and combined the 3D fracture mapping technique established by Ni et al. (Ni et al., 2021) and Xie et al. (Xie et al., 2020). All fracture virtual segmentation, reconstruction, and reduction procedures were performed under the supervision of an experienced orthopedic surgeon (W.W.). Initially, the raw CT scan data (DICOM 3.0 format) of the patient with a distal femur fracture was imported into E−3D Digital Medical Modeling and Planning System 18.02 (http://e3d-med.com), and the combined “Volume Rendering Segmentation” and “Intelligent Segmentation” function was utilized to extract and reconstruct each virtual fracture fragment and record the corresponding volume. Next, the fracture reduction function in the “Surgery Planning” module was selected to complete the virtual reduction of each fracture fragment. The fracture model is then aligned with the template in transparent mode, and the “Model Spacing Measurement” function of the “Measurement and Analysis” module is used to measure the distance between the fracture model and the template to ensure that the maximum distance between the models is within 5 mm. If the distance between the models is too great, use the “Fracture Reduction” function to fine-tune the models’ positions until the distance between them is acceptable. Finally, adjust the appropriate transparency and use the free curve to copy the outline of each fracture fragment along the surface of the template in 3D view, which is the process of depicting the fracture line, as shown in Figure 1. Moreover, the fracture area less than 1 cm^3^ in volume (Molenaars et al., 2015) which was determined as comminution zones was extracted by free curves using the “Surface Extraction” function, as shown in Figure 1F. In order to make our works more reproducible, we have included the detailed fracture mapping steps in the Supplementary Materials (Supplementary Figures S1–S14). After all the fracture lines were drawn and the comminuted regions were extracted, the “Fracture Statistical Analysis” function was used to create a heat map based on the spatial frequencies of the fracture lines and comminuted regions, as shown in Figures 2–7.

The template was reconstructed using thin-section CT data of the left femur of a 28-year-old healthy male. The total femur length of the template was 439 mm, and the maximum width between the medial and lateral distal femoral condyles was 82 mm. To further quantify the distal femoral fracture comminution region, we also recorded the number of fracture fragments and the corresponding volume of each fracture fragment in the distal femoral fracture comminution region of each patient, as well as the average volume of fracture fragments in this region.

### Statistical analysis

Quantitative data conforming to a normal distribution are expressed as mean ± standard deviation (SD); otherwise, they were expressed as median (interquartile range [IQR]). Normality was assessed using the Shapiro-Wilk test. The homogeneity of variance in groups was determined using the Levene’s test. If the variances between two normally distributed quantitative variables were equal, the unpaired two-sample *t*-test was used to compare the differences; if not, the Welch’s *t*-test was employed. For non-normally distributed quantitative data, differences were assessed using the Mann-Whitney *U* test. The qualitative data were expressed as frequencies and percentages. The Chi-square test or Fisher’s exact test was chosen for analysis of variance based on sample size and expected frequency in any cell, i.e., when more than 20% of cells have expected frequencies less than 5, the Fisher’s exact test was applied. *p* values less than 0.05 were considered statistically significant. All statistical analyses were completed using R software (version 4.1.0).

## Results

### Demographic and fracture characteristics

Among the 74 patients included in the study, the median age was 63 years (IQR, 25), with a mean body mass index (BMI) of 23.9 ± 3.1 kg/m^2^. Males constituted 54.1% (40 patients) of the population, and 56.8% (42 patients) sustained injuries on the left side. AO/OTA 33A distal femoral fractures were identified in 71.6% (53 patients) of the cases. As for the causative events leading to the fractures in our cohort, the majority resulted from slips (51.4%, 38 patients), followed by motor vehicle accidents (28.4%, 21 patients), falls from height (13.5%, 10 patients), and crush injuries (6.8%, 5 patients).

**FIGURE 8:**
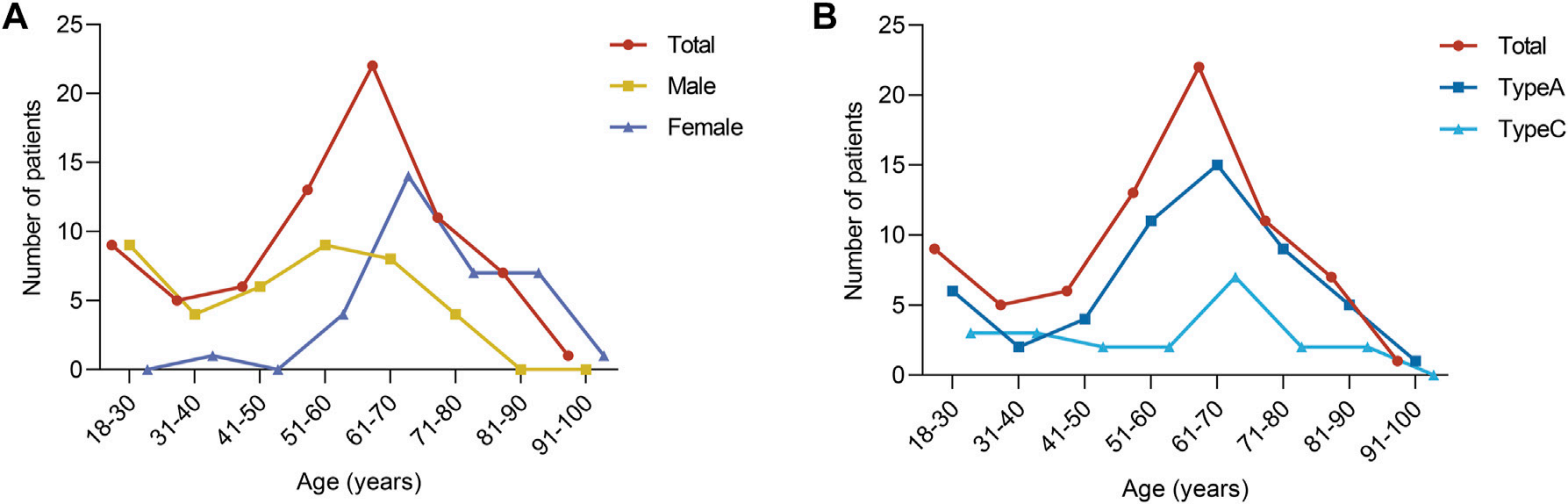
Frequency distribution of distal femoral fractures. **(A)** Distribution after grouping by age and sex. **(B)** Distribution after grouping by age and fracture classification.

Figure 8A shows the frequency distribution of distal femoral fractures grouped by age and gender. Distal femoral fractures were likely more common in males when the patient’s age was less than or equal to 50 years and were more evenly distributed across the age groups. When patients are older than 50 years, the number of female patients increases, with a peak in the age group of 61–70 years. Coincidentally, both AO/OTA 33A and 33C fractures are also predominant in the 61–70-year age group and are similarly distributed, as shown in Figure 8B.

In Table 1, patients with type 33A fractures were similarly distributed to patients with type 33C fractures in terms of age, BMI, sex, side of injury, and injury mechanism, with no significant differences (all *p* values greater than 0.05), while there were significant differences in terms of fracture fragment count, comminuted zone fracture fragment count, and mean comminuted zone fracture fragment volume (all *p* values less than 0.05), as shown in Table 2. The 33C fractures had more fracture fragments and more comminuted zone fracture fragments. The number of fractures in the comminuted zone was greater in the type 33C than the type 33A (all *p* values less than 0.05), while the volume of fractures in the comminuted zone was smaller in the type 33A than the type 33C (*p* = 0.029) (Table 2).

**TABLE 1:** Demographic and clinical characteristics of the research sample.

	AO/OTA classification		
	33A (*n* = 53)	33C (*n* = 21)	*p*-value
Age^a^, years	64 (20)	62 (25)	0.343
Height^a^, m	1.68 (0.15)	1.70 (0.14)	0.065
Weight^b^, kg	65.4 ± 10.0	65.4 ± 10.5	0.998
BMI^b^, kg/m^2^	24.2 ± 3.1	23.3 ± 3.0	0.263
Gender^c^			0.059
Male	25 (47.2)	15 (71.4)	
Female	28 (52.8)	6 (28.6)	
Side^c^			0.574
Left	29 (54.7)	13 (61.9)	
Right	24 (45.3)	8 (38.1)	
Injury mechanism^c^			0.142
MVA	13 (24.5)	8 (38.1)	
Slip	31 (58.5)	7 (33.3)	
Fall	5 (9.4)	5 (23.8)	
Crushing	4 (7.5)	1 (4.8)	

^a^ Values are expressed as Median (IQR).

^b^ Values are expressed as Mean ± SD.

^c^ Values are expressed as Frequency (Percentage).

**Abbreviations:** AO/OTA, Arbeitsgemeinschaftfür Osteosynthesefragen Foundation and the Orthopaedic Trauma Association; BMI, body mass index; MVA, motor vehicle accident; IQR, interquartile range; SD, standard deviation.

**TABLE 2:** Fracture characteristics.

	AO/OTA classification		
	33A (*n* = 53)	33C (*n* = 21)	*p*-value
No. of fracture fragments^§^	3.96 ± 2.81	8.24 ± 5.97	<0.001^*^
Comminuted fracture^†a^			0.059
Yes	33 (62.3)	8 (38.1)	
No	20 (37.7)	13 (61.9)	
No. of fracture fragments^§b^	0.92 ± 1.94	3.33 ± 4.67	0.008^*^
Average volume of fracture fragments^§b^, cm^3^	0.14 ± 0.22	0.27 ± 0.24	0.029^*^

^a^ Defined as containing a fracture fragment of less than 1 cm^3^ in volume.

^b^ Refers to a fracture fragment of less than 1 cm^3^ in volume. †Values are expressed as Frequency (Percentage). §Values are expressed as Mean ± SD. *Results differ significantly by *t*-test or Chi-square test.

**Abbreviations:** AO/OTA, Arbeitsgemeinschaftfür Osteosynthesefragen Foundation and the Orthopaedic Trauma Association; SD, standard deviation.

### Fracture maps

All fracture lines and comminuted areas were extracted and pooled in the template, respectively, as shown in Figures 2, 3. The fracture line distribution heat map indicated that most of the heat zones were in the femoral epiphysis, intercondylar notch of the femur, and patellofemoral joint. In these regions, the highest incidence of fracture lines was around 35.1% (26/74). In addition, the majority of fracture lines follow the distal femur from superior to inferior and extend to the articular surface, forming a “Y” shape. The comminuted area distribution heat map suggests that the fracture regions are mainly located in the lateral, anterior, and posterior aspects of the femoral diaphysis, with less involvement on the medial side. The maximum frequency of these three regions was 10.8% (8/74). The frequency of each comminuted zone was nearly centripetal, i.e., the more frequent regions were clustered in the lateral, anterior, and posterior femoral epiphyseal centers.

As illustrated in Figures 4, 5, the fracture lines and comminuted areas of OA/OTA 33A fractures are mainly located in the area around the femoral epiphysis. The fracture line heat map suggests that the fracture line is most concentrated on the anterior surface of the supracondylar region of the femur, where the maximum value of fracture line occurrence is around 47.2% (25/53). The fracture line heat zones on the medial femoral column were more dispersed than the lateral column. According to the scale of the template model, the spacing of the medial hot zones was approximately 8 cm, whereas the spacing of the lateral hot zones was approximately 2.5 cm. Most fracture lines remain in the medial area of the condyle from approximately 15 to 4.5 cm distal to the lateral side of the joint line. The densest portions of all three regions were distributed centripetally. The comminuted area heat map indicates that most comminuted zones are found on the anterior and posterior surfaces of the supracondylar femur, as well as the lateral inferior region. The outer inferior region was more densely packed, with the anterior side dispersed and the posterior side clustered in a triangular arrangement, with a maximum frequency of 11.3% (6/53) and a separation of roughly 6 cm.

In the 21 AO/OTA 33C distal femoral fractures, the fracture lines were mostly clustered at the femoral epiphysis and the intercondylar notch, as shown in Figure 6. The heat map shows a “Y”-shaped area of dense fracture lines. The hot zone of the intercondylar notch forms the lower part of the “Y”. The hot zone of the femoral epiphysis extends from the patellofemoral joint upwards to the lateral and medial aspects of the epiphysis, respectively, forming the upper part of the “Y”. This is also consistent with the AO/OTA classification description (Meinberg et al., 2018). Of these areas, the anterior and posterior surfaces of the femoral epiphysis, the medial and intercondylar notches have the greatest fracture line density. Relatively infrequently, fracture lines on the lateral surface of the femoral epiphysis occur. According to the scale of the template, the distance from the lower edge of the medial hot zone to the joint line was approximately 4 cm, the distance from the upper border to the joint line was approximately 10 cm, and the vertical distance between the upper border and the lower border was approximately 5.5 cm. In Figure 7, the heat map of the comminuted area shows that the comminuted area is mainly concentrated on the anteromedial aspect of the femoral diaphysis and that the densest area remains close to the midline of the anterior surface of the diaphysis, with approximately 3 cm from the lower edge of the hot zone to the joint line, approximately 7 cm from the upper boundary to the joint line, and a vertical distance of approximately 4 cm between the upper and lower edges.

## Discussion

Using a 3D CT-based fracture mapping approach, we retrieved fracture lines and comminuted regions from 53 AO/OTA 33A and 21 33C distal femoral fractures in this retrospective analysis. Compared to 2D CT-based fracture mapping, this technique provides a more accurate delineation of the fracture morphology (Dugarte et al., 2018). For orthopedic surgeons to formulate preoperative planning and internal fixation strategies, accurate and realistic fracture morphology is necessary. So, we summarized the morphological features of distal femoral fractures based on heat maps of fracture lines and comminuted regions to represent some useful information in this respect.

From the 33C distal femoral fracture line heat map, we found that the fracture lines were primarily concentrated in the femoral diaphysis and intercondylar notch, and that the diaphysis was involved in both the medial and lateral columns, suggesting that in some cases, additional medial column support may be required. In recent years, double plates have been used to treat several complex distal femoral fractures with severe comminution or osteopenia. Studies (Steinberg et al., 2017; Bologna et al., 2020) have demonstrated that the insertion of a medial plate improves radiological and clinical outcomes. Some biomechanical study has also shown that medially placed plate support reverses the loads with inversion of the lower limb by sharing some of the stresses of the lateral plate (Zhang et al., 2018), hence enhancing fracture stability (Park et al., 2019). According to the 33C fracture line heat map, most intercondylar fracture lines go back through the intercondylar notch to the area near the anterior cruciate ligament (Ferretti et al., 2007). This suggests that orthopedic surgeons should pay special attention to the presence of concurrent ligamentous injuries when examining and exploring patients with distal femoral fractures during surgery.

According to the 33C fracture comminution area distribution heat map, the comminution zone is mostly located on the anteromedial aspect of the diaphysis, which indicates that the orthopedic surgeon should consider the median surgical approach as an alternative approach to expose the femoral diaphysis, such as the “Swashbuckler” approach described by Starr et al. (Starr et al., 1999). Combining the heat map of the 33C fracture line and the comminuted area, we found that the comminuted area of this fracture is less likely to involve the lateral and posterior medial aspects of the distal femur. This suggests that when treating these fractures with double plate fixation, the medial plate may need to be placed slightly more posteriorly or with a bridging approach, thus avoiding these comminuted areas. At present, we are not aware of a specifically designed, uniform medial plate for the treatment of type 33C fractures. These characteristics of the fracture line and comminuted area distribution may inform the design of future internal fixation devices.

In the investigation of the fracture line and comminuted region for 33A distal femoral fractures, the fracture pattern of the supracondylar section of 33A and 33C fractures was found to be distinguishable. The majority of fracture lines and comminuted areas in type 33A distal femoral fractures are oblique, extending from medial superior to lateral inferior along the distal end of the femur. In 33C distal femoral fractures, the fracture lines in the supracondylar region curve from lateral superior to medial inferior and at a less oblique angle than in 33A fractures. This implies that different types of fractures should be distinguished and fracture internal fixation protocols should be used. When fixing a 33A distal femoral fracture with a lateral plate, fixation across a dense area of the fracture line may need to be considered. Moreover, the more widespread lateral fracture line of 33A distal femoral fractures suggests that, when utilizing a single plate for lateral fixation, the plate length should be suitable and may need to be at least 15 cm or more from the joint line.

When comparing the volume of the fracture fragments in the comminution area between the two fracture types, we discovered that the 33A distal femoral fractures had a smaller volume (*p* = 0.029), which indicates that complex 33A distal femoral fractures appear to be more susceptible to bone loss and that bone grafting should be considered if necessary. Furthermore, all regions of high-density comminution in 33A distal femoral fractures were distributed centripetally. This may be a result of the patient’s sustaining a high-energy injury and the violence propagating axially.

This research could potentially aid in the refinement of finite element models for distal femoral fractures. The osteotomy schemes for complex 33A and 33C distal femoral fracture finite element models remain controversial. Park et al. (Park et al., 2019) attempted to imitate a 33A3 fracture by establishing an osteotomy gap that was 2.5 cm wide and 6 cm from the distal femoral articular line. Wright et al. (Wright et al., 2020) created a 2 cm wide osteotomy gap 6.5 cm from the intercondylar notch of the femur to replicate the same type of fracture. Zhang et al. (Zhang et al., 2018) mimicked a 33C2 fracture by establishing an osteotomy gap 1.5 cm wide and 6.5 cm away from the joint line. Duffy et al. (Duffy et al., 2006) modeled a 33C3 fracture by producing a 1 cm wide supracondylar femoral fracture gap 6 cm proximal to the joint line. In both finite element analyses of 33C distal femoral fractures, the researchers simulated intra-articular fractures by cutting the intercondylar femoral notch directly in the sagittal plane.

However, in the current investigation, the 33A distal femoral fracture comminution area heat map revealed that most of the comminution area began roughly 4 cm from the distal joint line and concluded approximately 10 cm from the joint line, all within a vertical distance of approximately 6 cm. In the comminution area heat map of 33C distal femoral fractures, the distance between the distal joint line and the lower edge for most of the comminuted region is around 3 cm, and the vertical length of the area is roughly 4 cm. Although the spacing of the comminuted region measured on the femoral template disregards inter-individual variability, the apparently narrow width of the osteotomy gap reported by the aforementioned authors, and thus the inability to accurately model fracture morphology, may compromise the validity of the results of these studies. So, we propose that for the 33A distal femoral fracture finite element model, the osteotomy site should begin 4 cm away from the joint line, and the osteotomy gap should be around 6 cm in height. For the 33C distal femoral fracture model, the osteotomy should begin 3 cm from the joint line, and the osteotomy gap should be close to 4 cm in height. A sagittal cut should be performed lateral to the intercondylar notch of the femur when imitating a comminuted portion of the articular surface. The resulting distal femoral finite element model may be more accurate and closer to reality than models generated in prior investigations.

So, the data and models derived from this study provide a comprehensive basis for constructing finite element models, which will undoubtedly prove to be an invaluable reference for future investigations in this field. This knowledge can guide and stimulate further research into innovative assessment techniques, treatment strategies, and preventive measures related to distal femur fractures. More specifically, our study offers critical insights into the surgical treatment of distal femur fractures, particularly those classified as types 33A and 33C. It presents valuable information regarding surgical access and internal fixation strategies. This can greatly enhance the decision-making abilities of surgeons, enabling them to select and optimize surgical techniques and approaches with a greater degree of confidence and knowledge. In turn, these informed decisions can potentially result in improved patient outcomes. For example, we might anticipate fewer complications and expedited recovery times.

There are several limitations to this study. First, the research only described fracture morphology and did not further explore its association with patient demographics or prognosis. The reconstruction and morphological analysis of distal femur fractures in this investigation were acquired from thin-section CT imaging data; thus, the findings have limited relevance and are not indicative of the fracture pattern in patients who did not have CT scanning. In addition, as research on AO/OTA 33B distal femoral fracture line maps has already been published, only 33A and 33C fractures were included in this study, which may have resulted in selection bias. Again, a uniform template was used in this study, which did not take into account individual differences between patients and may have some discrepancies between the true values. Finally, this single-center, retrospective study included a small sample size due to the low incidence of distal femoral fractures. However, sample size, different regions, and different ethnicities may all be potential influencing factors (Everhart et al., 2016) and should be considered in future studies.

## Conclusion

The fracture line and comminuted area models demonstrate that the features of AO/OTA 33A and 33C distal femur fractures are distinct. In 33A distal femoral fractures, the fracture line and comminuted area are less frequently involved medially, indicating that external locking plate fixation can adequately support most fractures. The 33C fracture line and comminution zone models show that the medial femoral epiphysis is frequently involved, suggesting that posterior medial placement of the plate or bridging fixation may be more appropriate when adding a medial plate to stabilize the medial column. In addition, the high frequency of comminuted areas in these fractures tends to be localized near the distal femoral axis, suggesting that a median approach may be a viable option for choosing an adequate surgical incision to expose the diaphysis. Finally, most current finite element studies lack homogeneous osteotomy criteria, have tiny osteotomy gaps, and have high osteotomy locations. This study presents partial osteotomy information derived from fracture line and comminuted area models. In the future, standard biomechanical trials and randomized controlled studies will be needed to further validate the clinical utility of the osteotomy and internal fixation strategies recommended in this research.

## Data Availability

The raw data supporting the conclusion of this article will be made available by the authors, without undue reservation.
