# Supplementary material for: Three-dimensional computed tomography mapping techniques in the morphometric analysis of AO/OTA 33A and 33C distal femoral fractures: a retrospective single-center study
Source: Front Bioeng Biotechnol. 2023 Jun 15;11:1162214. doi: 10.3389/fbioe.2023.1162214 (PMC10311492; doi:10.3389/fbioe.2023.1162214)
Supplement: Supplementary file 1 [file DataSheet1.DOCX]

**Three-dimensional computed tomography mapping techniques in the morphometric analysis of AO/OTA 33A and 33C distal femoral fractures: A retrospective single-center study**

**Details of Three-Dimensional (3-D) Mapping Technique**

**1. Obtain Computed Tomography (CT) Data.**

The DICOM (Digital Imaging and Communications in Medicine)-formatted CT data of all distal femoral fractures was acquired from the PACS (picture archiving and communication system) workstation.

**2.** **Schematic of Fracture Mapping.**

(1) Import the raw CT scan data (DICOM 3.0 format) of the patient with a distal femur fracture into E-3D Digital Medical Modelling and Planning System 18.02 (http://e3d-med.com).

(2) Create a mask to indicate which part to reconstruct by using the "Volume Rendering Segmentation" function (Figure S1). At this moment, the mask contains the distal femur, patella, tibia, and fibula (Figure S1A).

(3) Remove parts of the patella, tibia, and fibula from the mask using the "Easy Seed Point" function in the "3-D" module (Figure S2).

(4) Before reconstructing the 3-D models, we split the mask that contains all the fragments into separate masks using the "Cluster Separation" function (Figure S3), and then we can begin to reconstruct the 3D models of each fragment.

(5) Reconstruct the 3-D models of each fragment using the "Solid Modelling" function and choose the "Optimal Quality" (Figure S4).

(6) Reduce the fragments to the proximal part using the “Fracture Reduction” function in the “Surgery Planning” module (Figures S5 and S6).
(7) Combined fracture fragments after reduction using the “Multi-model Merging” function in the “CAD Modelling” module, and then select the “Stabilization Method” and set the “Precision” to a maximum of 10 (Figure S7).

(8) Mirror the fracture model to match the prepared temple model using the “Mirror Restoration” function in the “Surgery Planning” module. Then click on the “Mirror Bilateral” and “Generate Model” buttons in turn to generate a mirror model (Figure S8).

(9) Import the temple model and set its transparency to 50% (Figure S9).

(10) Superimpose the fracture model to fit the standard template using the “Fracture Reduction” function in the “Surgery Planning” module (Figure S10).

(11) Measure the distance between the fracture model and the template model using "Model Spacing Measurement" in the "Measurement Analysis" module (Figure S11). Then continue to adjust the position between the two models based on the results.

(12) Draw fracture lines on the template model using the “Curve” and “Model Surface Free Curve” functions in the "CAD Modelling" module (Figures S12 and S13).

(13) Extract the comminution area using the “Surface Offset” function in the “CAD Modelling” module (Figure S14).

(14) Repeat steps (1) to (13) above.


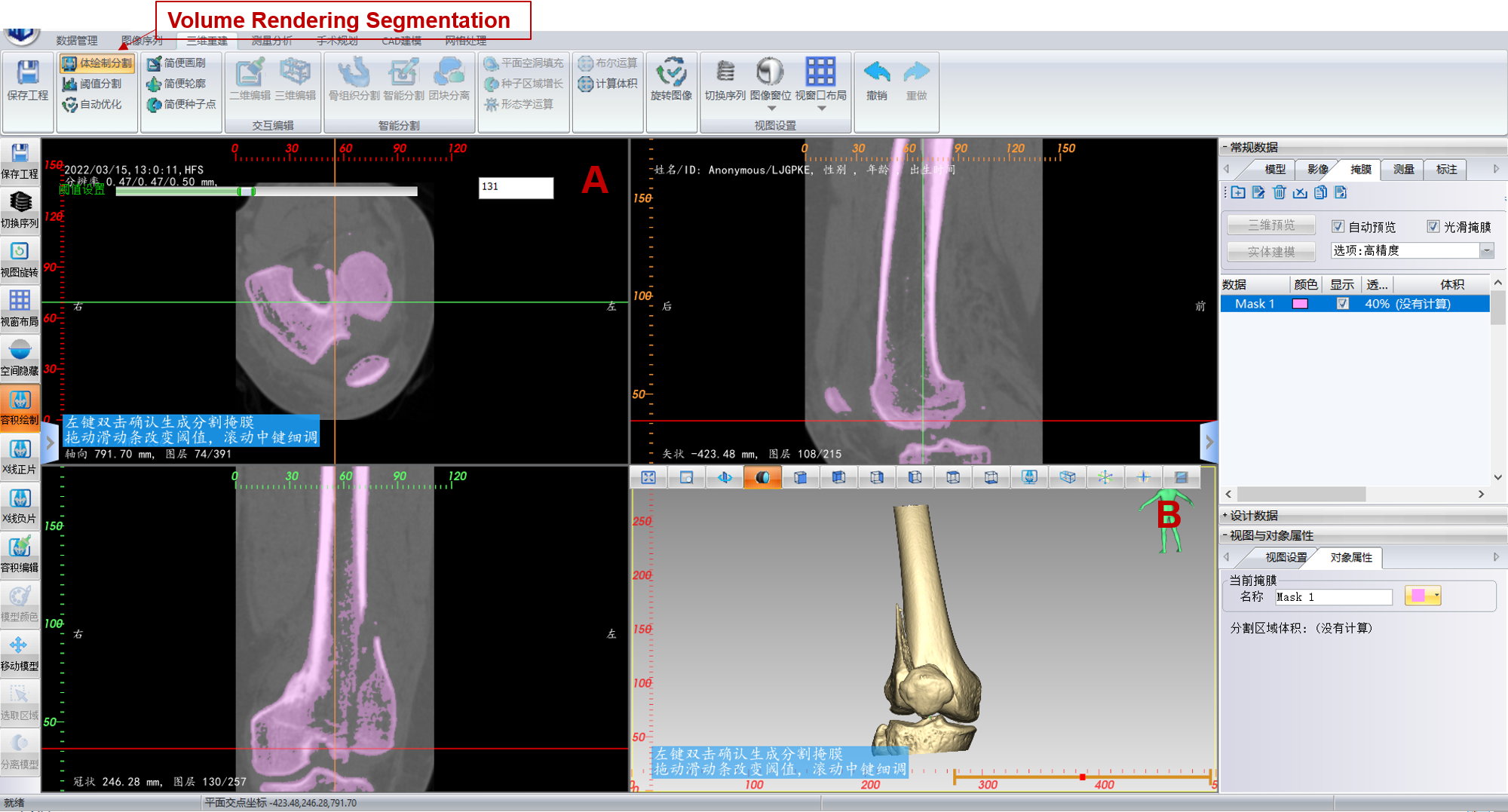


**Figure S1.** Create a mask to indicate which part to reconstruct. (A) Set the optimal threshold value. (B) Volumetric rendering with real-time imaging according to the selected threshold value.

**
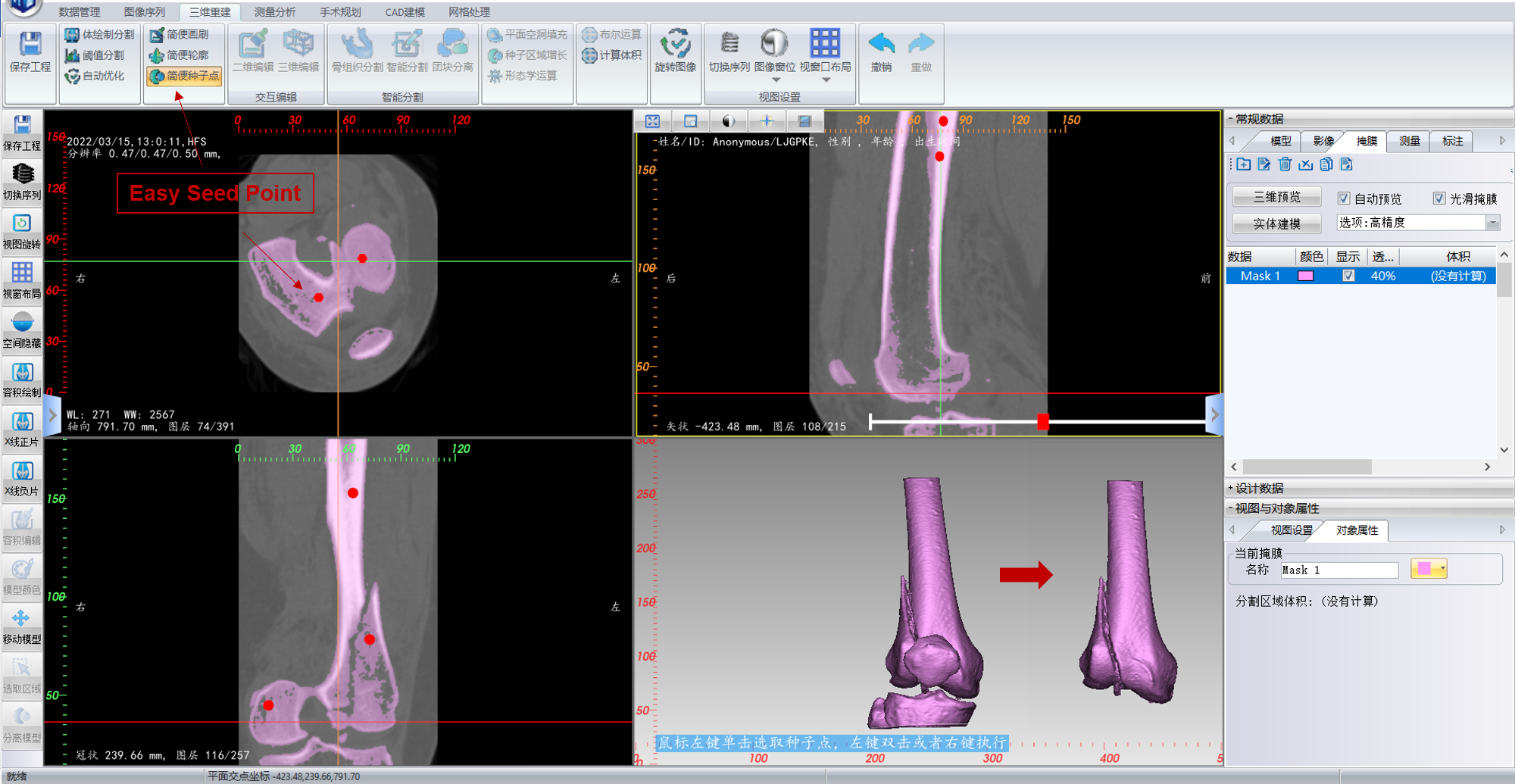
**

**Figure S2.** Remove parts of the patella, tibia, and fibula from the mask using the “Easy Seed Point” function.

**
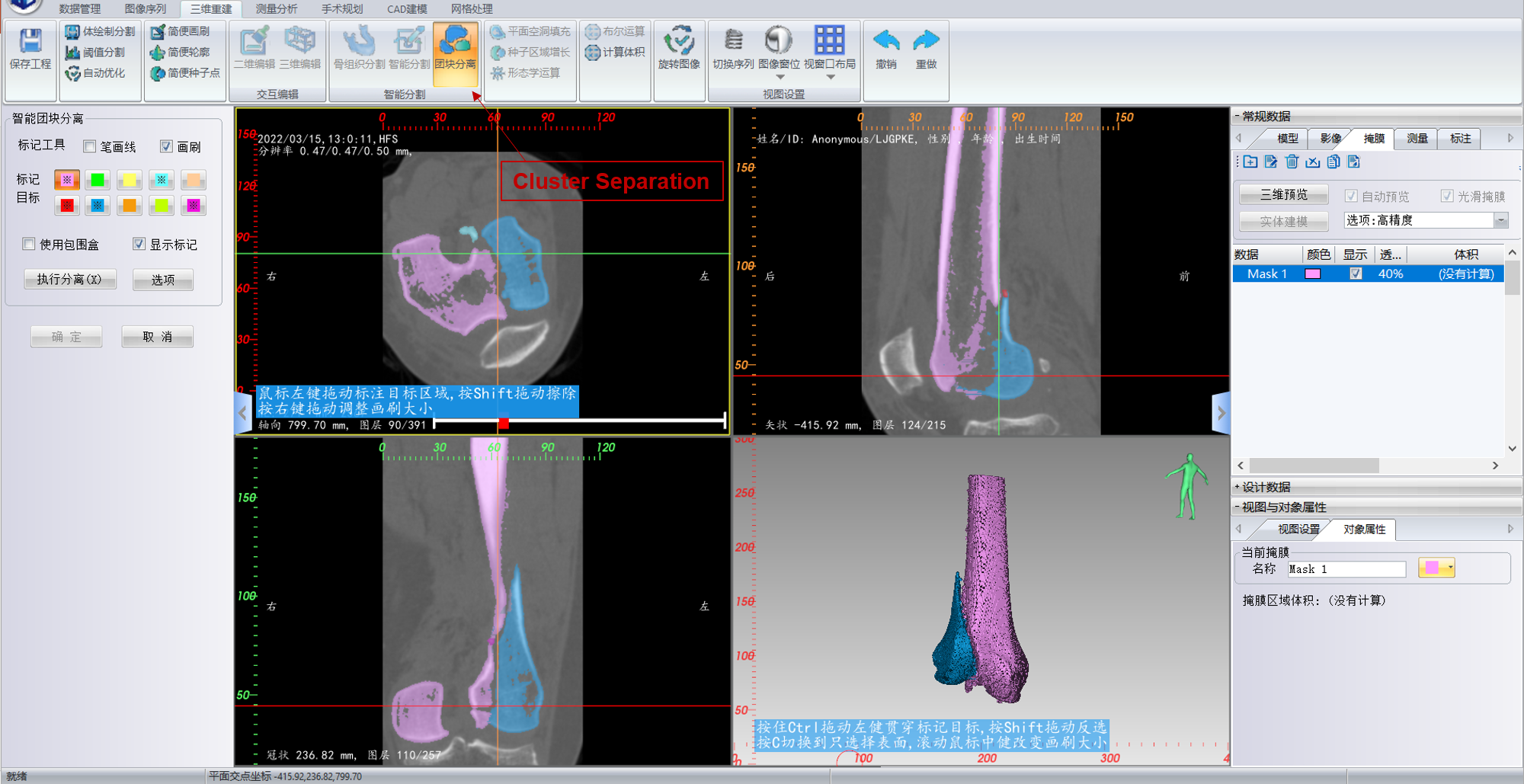
**

**Figure S3.** Split the mask that contains all the fragments into separate masks using the “Cluster Separation” function.

**
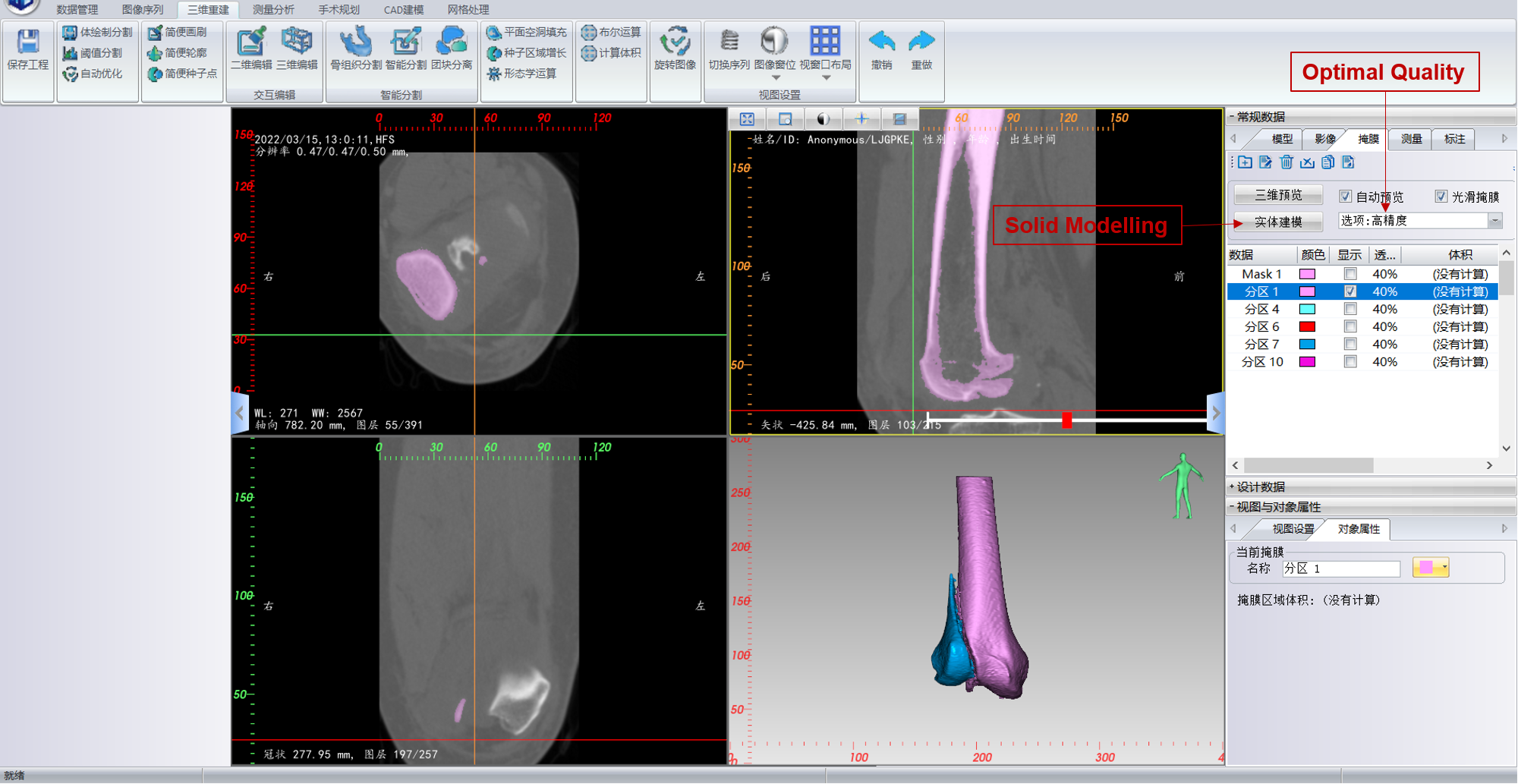
**

**Figure S4.** Reconstruct the 3-D models of each fragment using the “Solid Modelling” function and choose the “Optimal Quality”. 3-D, three-dimensional.

**
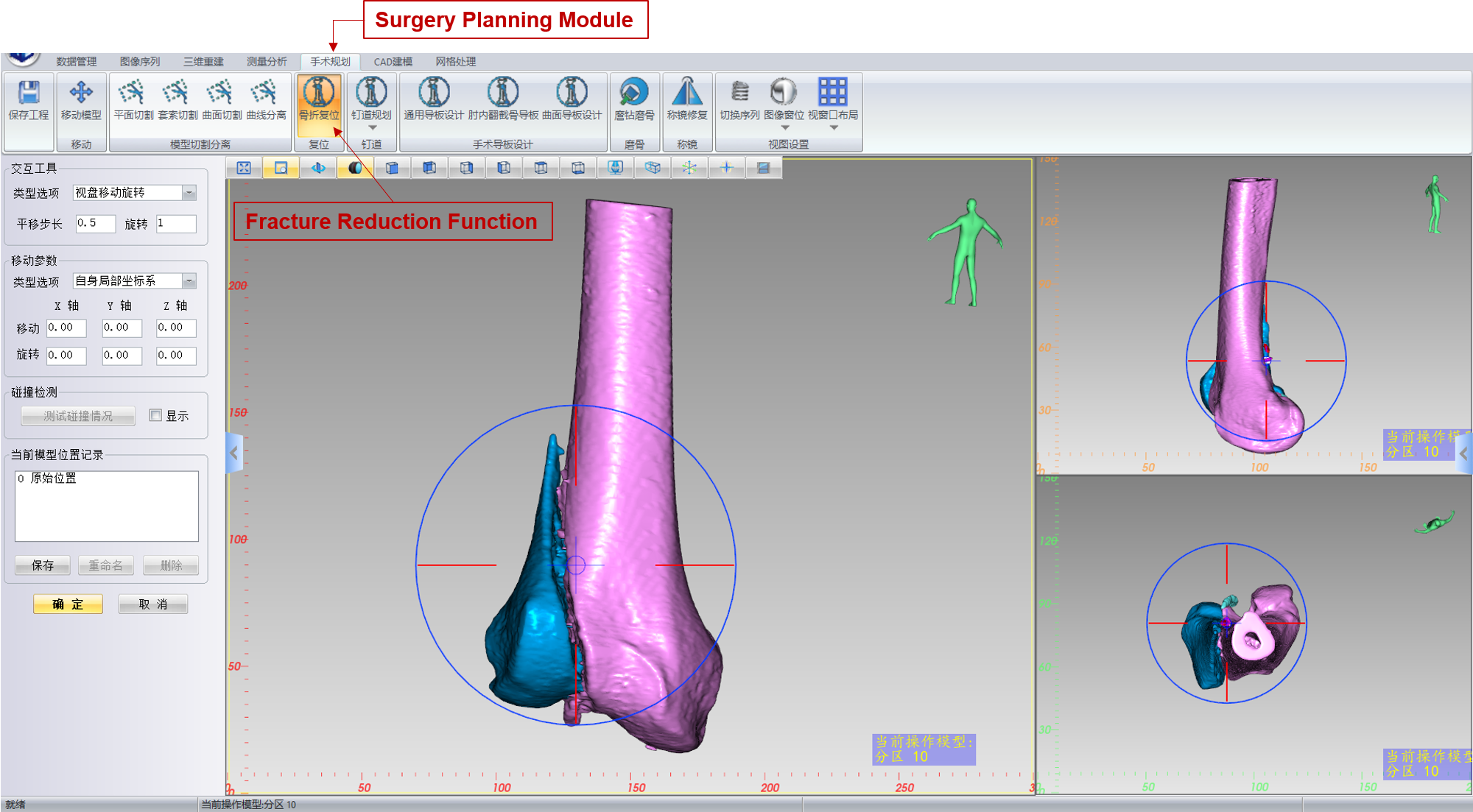
**

**Figure S5.** Reduce the fragments to the proximal part using the “Fracture Reduction” function in the “Surgery Planning” module.

**
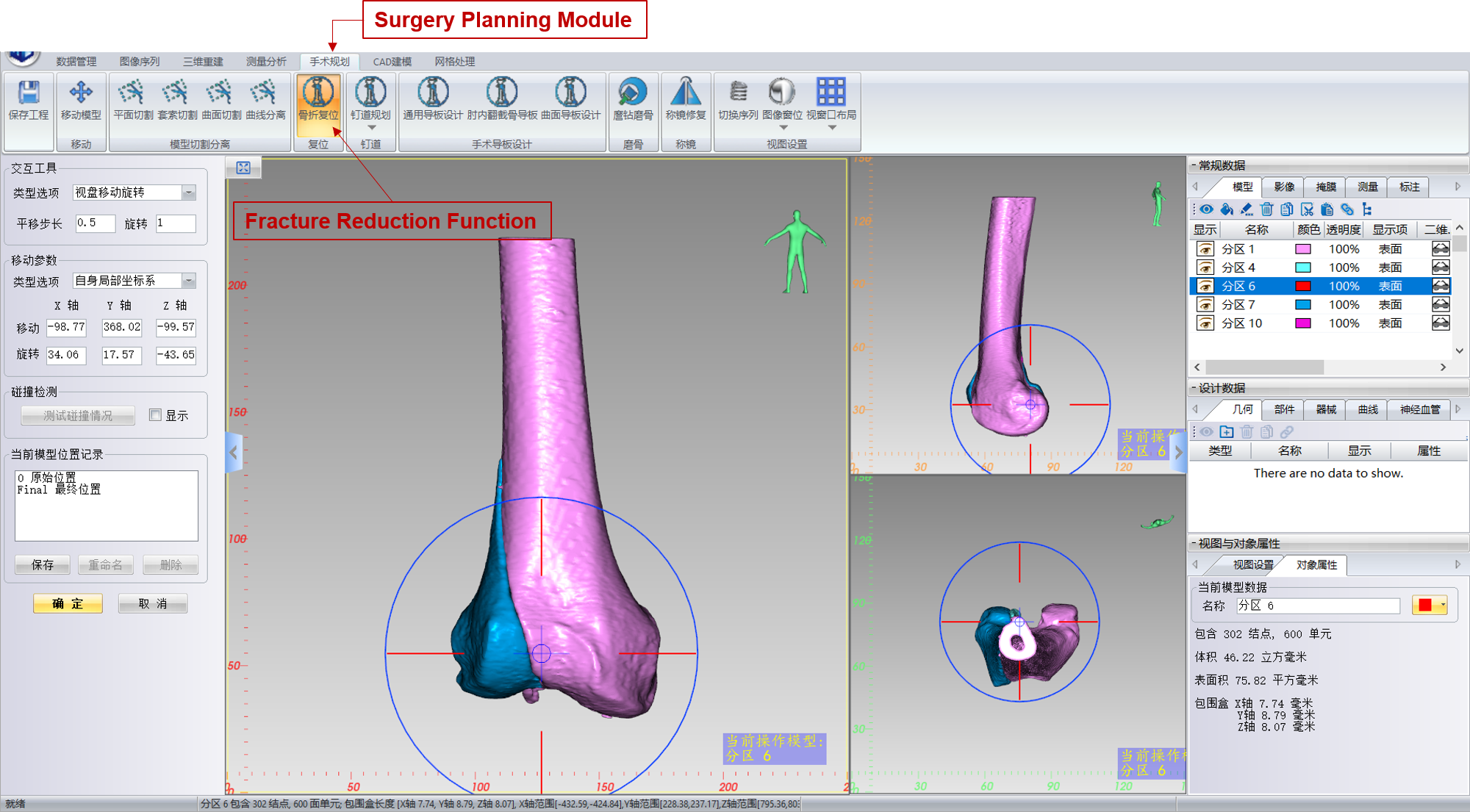
**

**Figure S6.** All fracture fragments have been reduced.

**
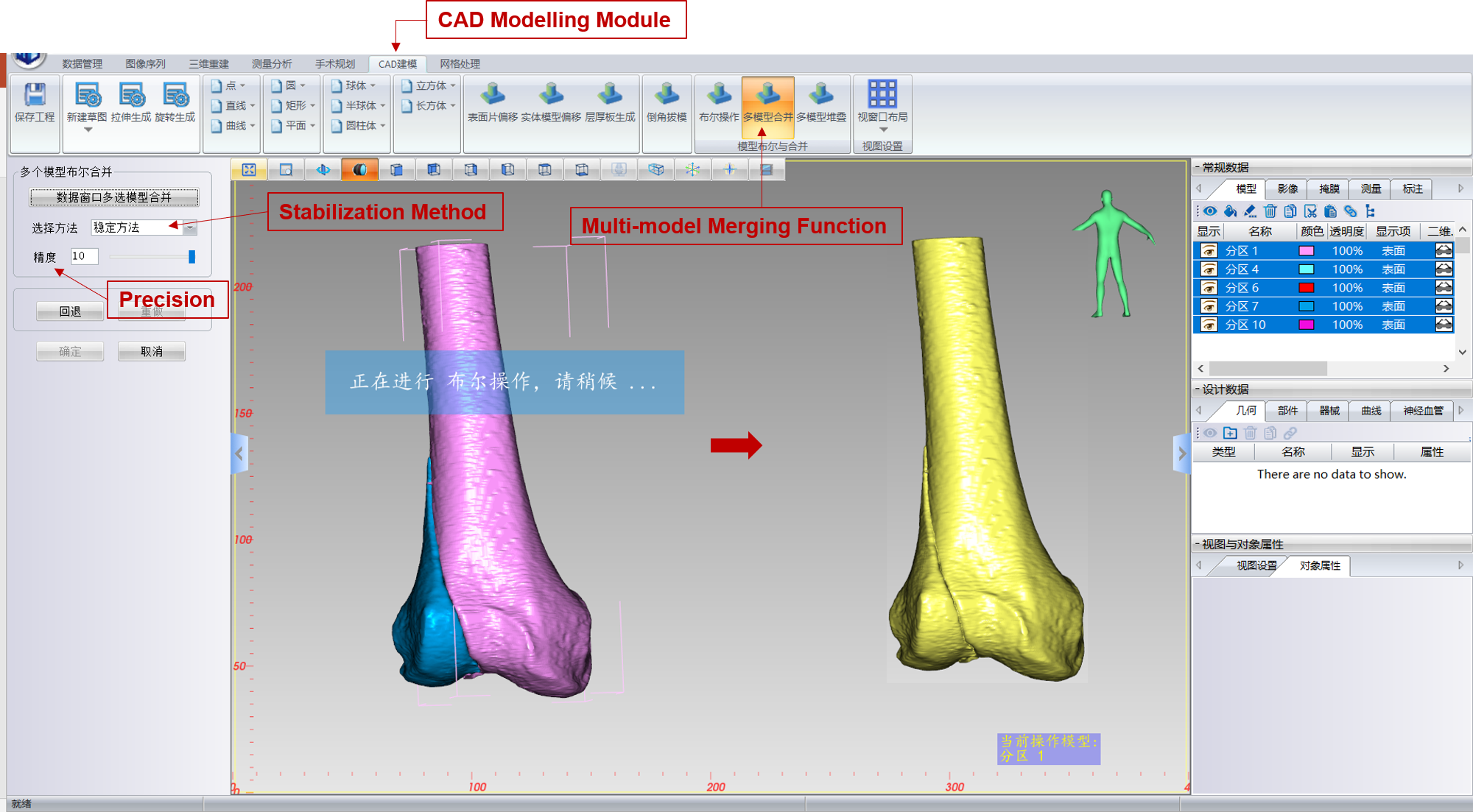
**

**Figure S7.** Combined fracture fragments after reduction using the “Multi-model Merging” function in the “CAD Modelling” module, and then select the “Stabilization Method” and set the “Precision” to a maximum of 10.

**
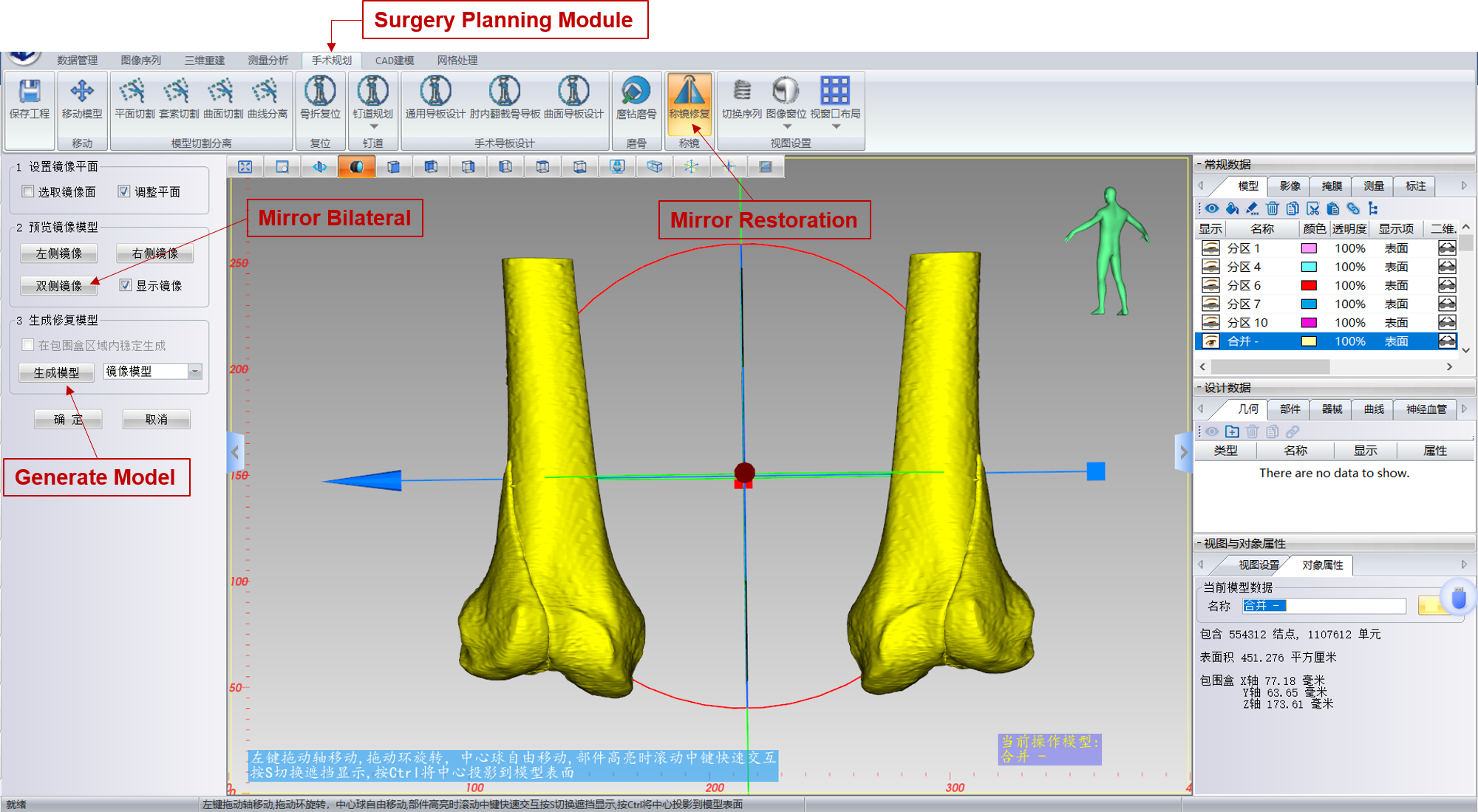
**

**Figure S8.** Mirror the fracture model to match the prepared temple model using the “Mirror Restoration” function in the “Surgery Planning” module. Then click on the “Mirror Bilateral” and “Generate Model” buttons in turn to generate a mirror model.

**
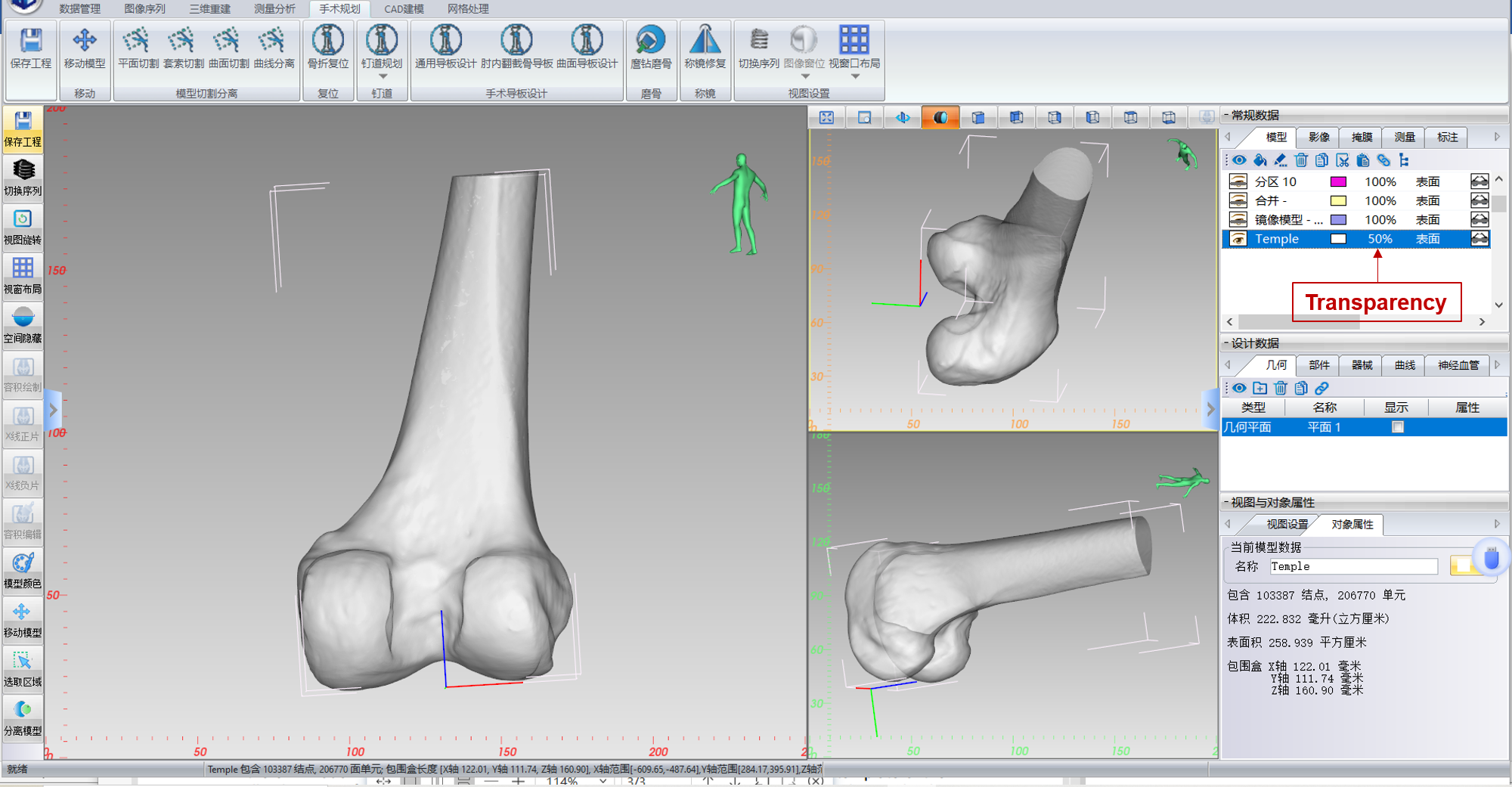
**

**Figure S9.** Import the temple model and set its transparency to 50%.

**
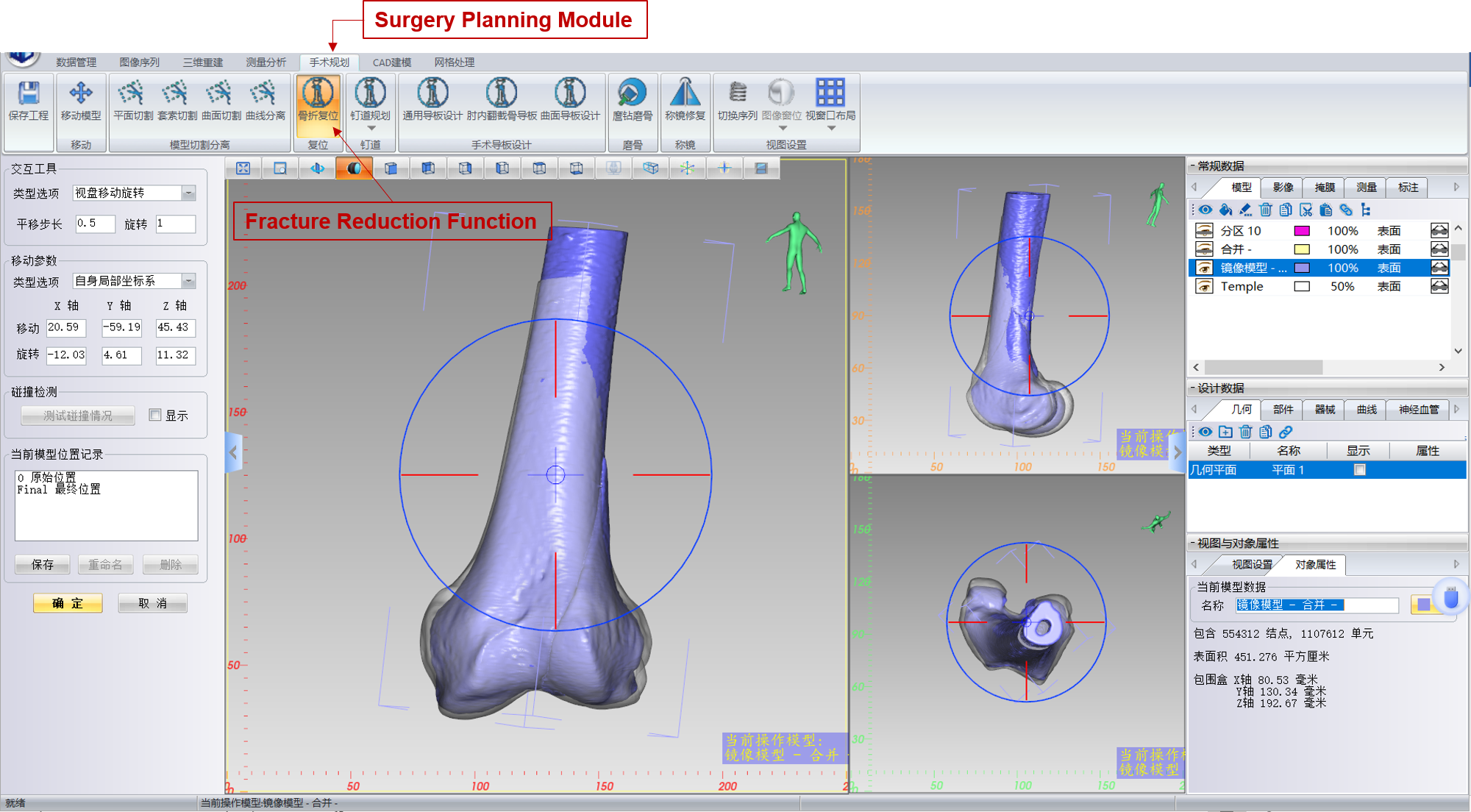
**

**Figure S10.** Superimpose the fracture model to fit the standard template using the “Fracture Reduction” function in the “Surgery Planning” module.

**
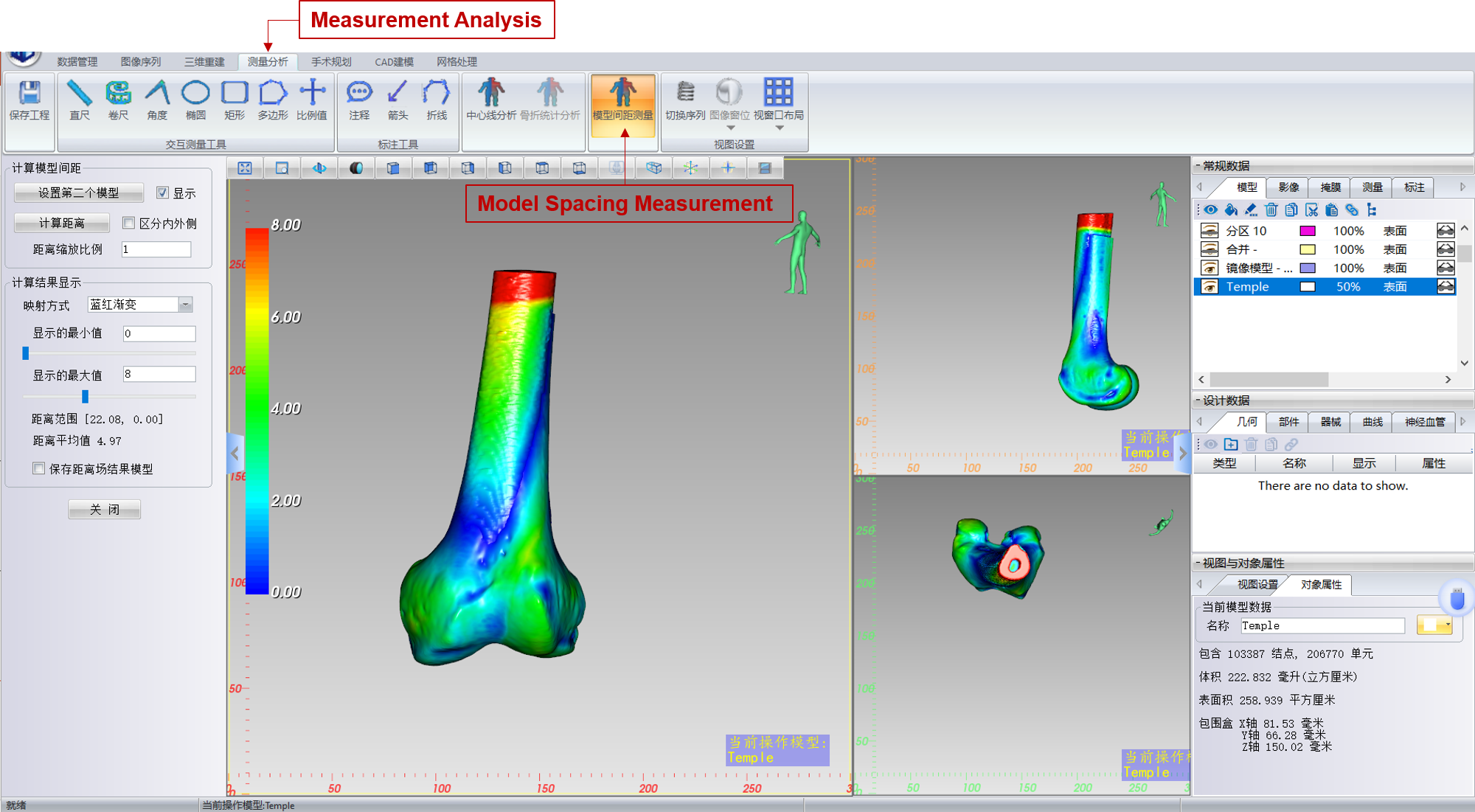
**

**Figure S11.** Measure the distance between the fracture model and the template model using “Model Spacing Measurement” in the “Measurement Analysis” module.

**
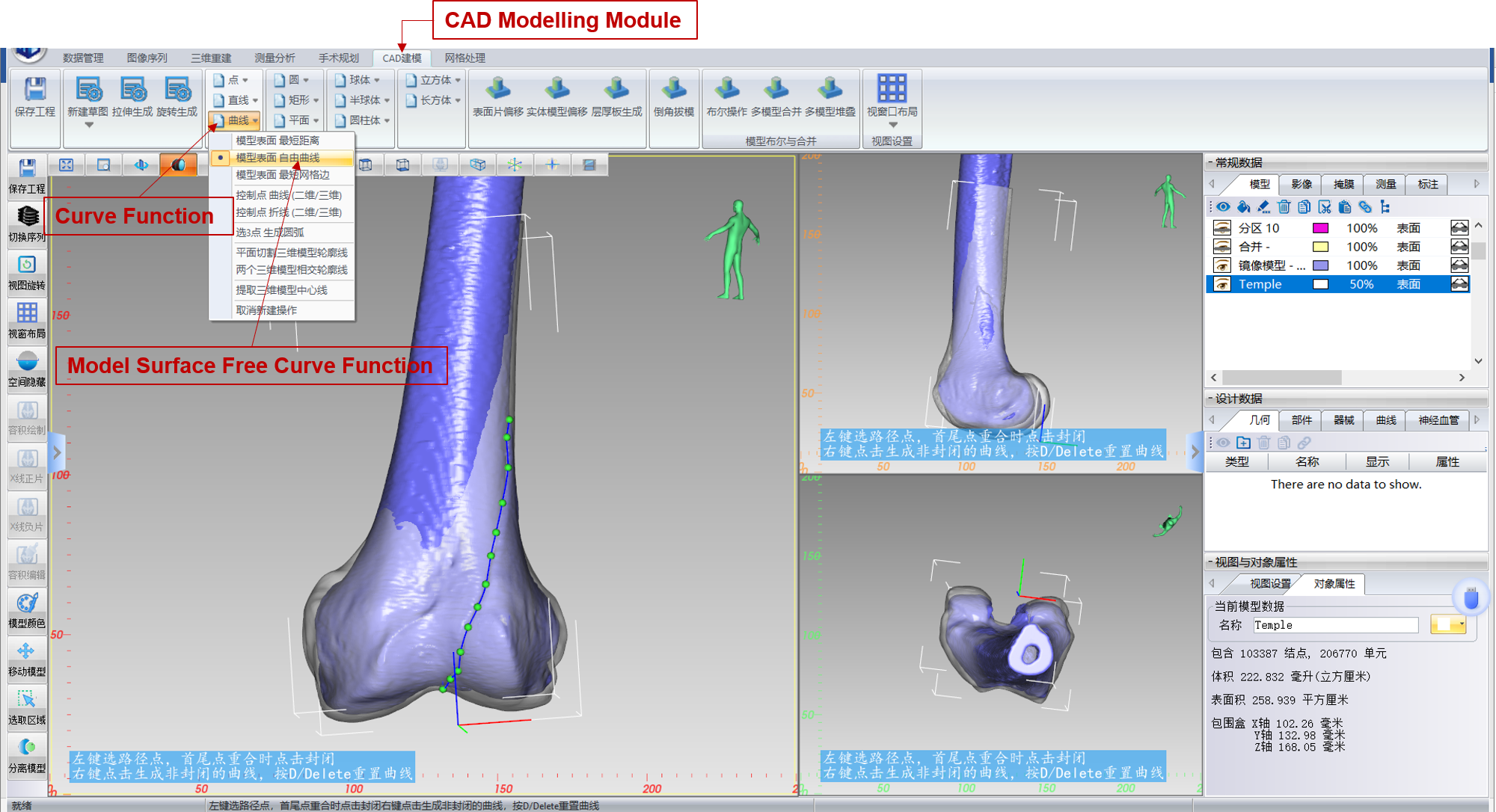
**

**Figure S12.** Draw fracture lines on the template model using the “Curve” and “Model Surface Free Curve” functions in the "CAD Modelling" module.

**
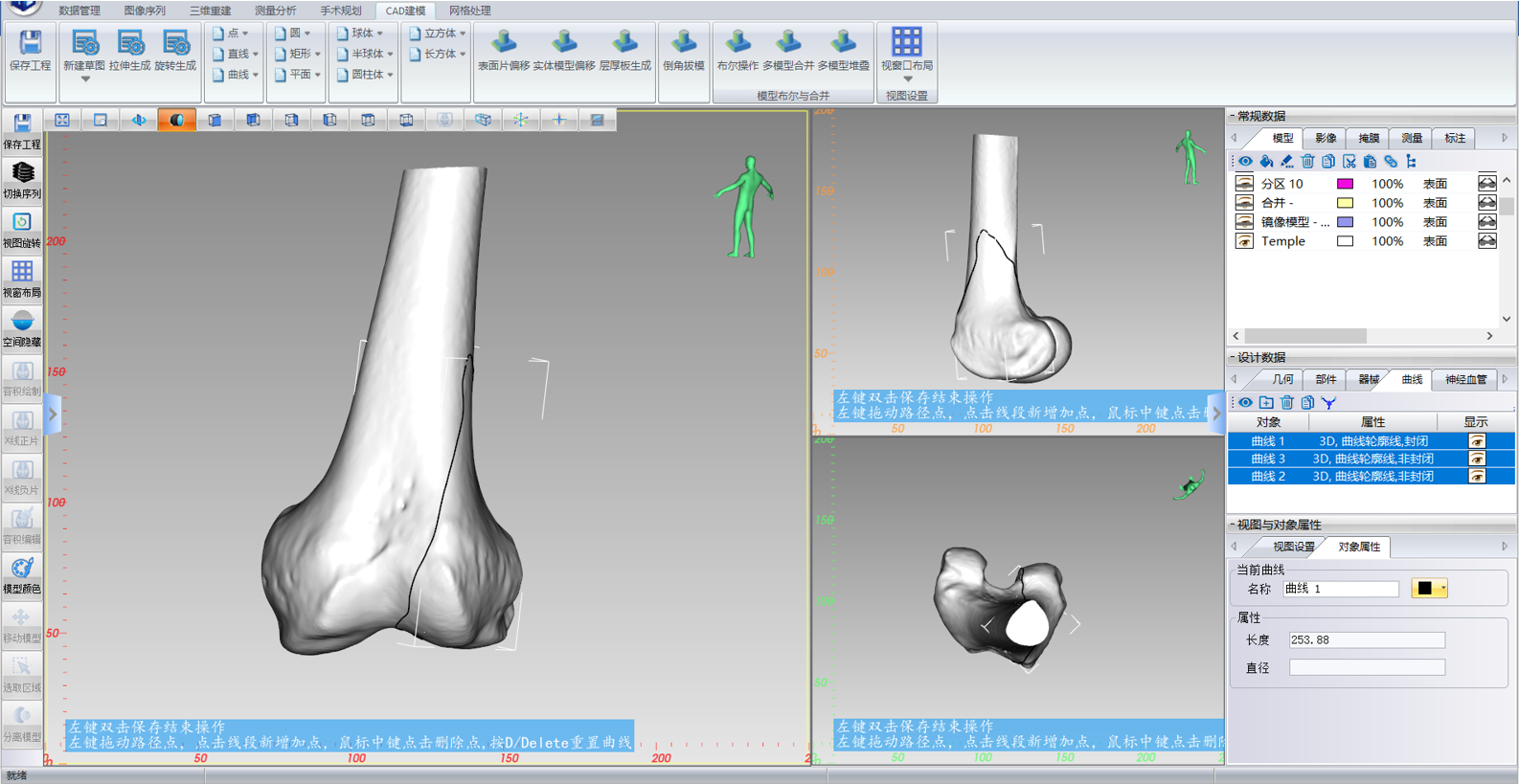
**

**Figure S13.** All fracture lines have been drawn.


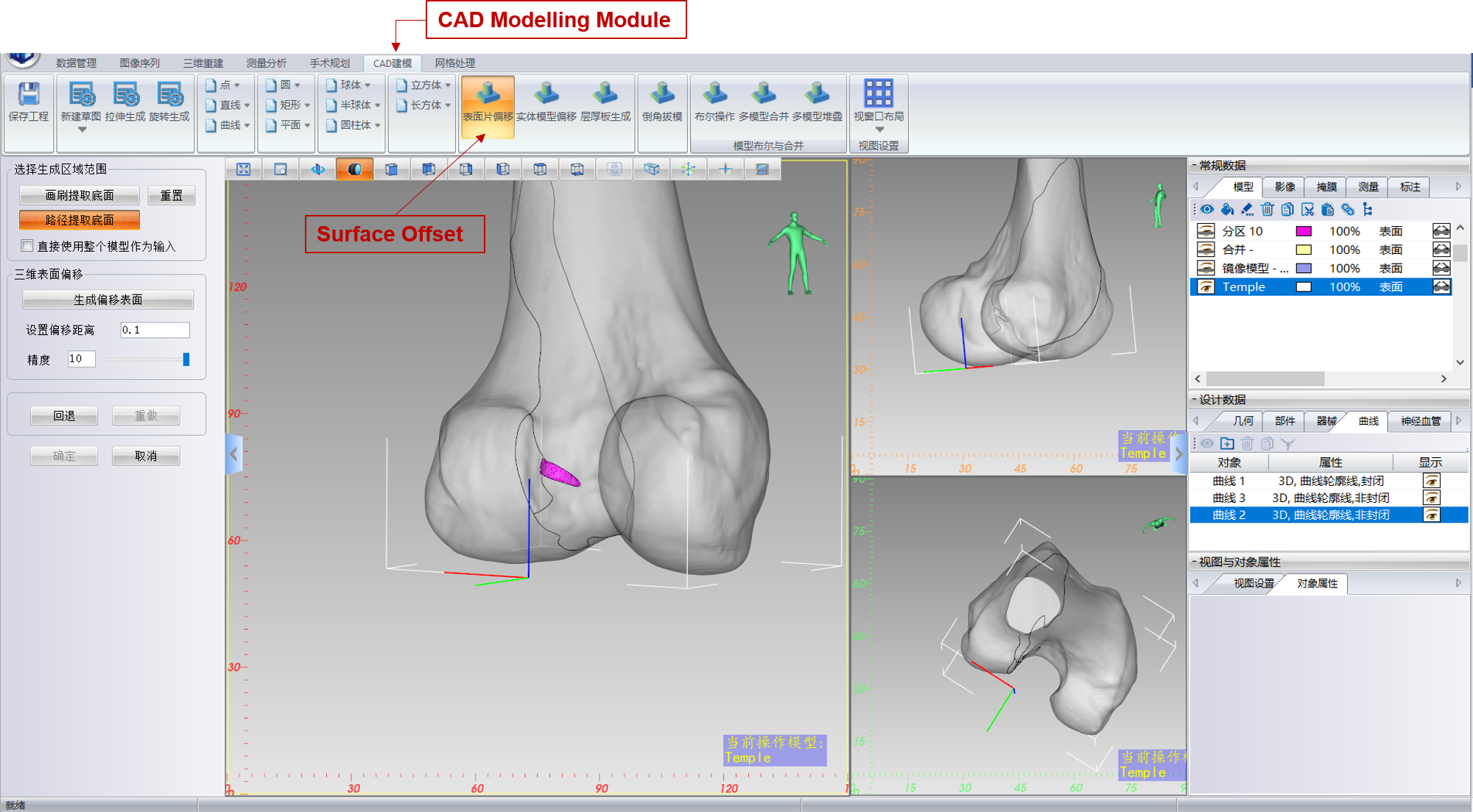


**Figure S14.** Extract the comminution area using the “Surface Offset” function in the “CAD Modelling” module.
